# Intestinal neutrophil extracellular traps promote gut barrier damage exacerbating endotoxaemia, systemic inflammation and progression of diabetic retinopathy in type 2 diabetes

**DOI:** 10.1007/s00125-024-06349-4

**Published:** 2025-01-28

**Authors:** Jason L. Floyd, Ram Prasad, Mariana D. Dupont, Yvonne Adu-Rutledge, Shambhavi Anshumali, Sarbodeep Paul, Sergio Li Calzi, Xiaoping Qi, Akanksha Malepati, Emory Johnson, Patricia Jumbo-Lucioni, Jason N. Crosson, John O. Mason, Michael E. Boulton, Robert S. Welner, Maria B. Grant

**Affiliations:** 1 Department of Ophthalmology and Visual Sciences, Heersink School of Medicine, University of Alabama at Birmingham, Birmingham, AL USA; 2 Pharmaceutical, Social and Administrative Sciences, Samford University, Birmingham, AL USA; 3 Retina Consultants of Alabama, Birmingham, AL USA; 4 Department of Medicine, Division Hematology/Oncology, Heersink School of Medicine, University of Alabama at Birmingham, Birmingham, AL USA

**Keywords:** Diabetes, Diabetic retinopathy, Gut permeability, NETosis, Neutrophil, PAD4

## Abstract

**Aims/hypothesis:**

Within the small intestine, neutrophils play an integral role in preventing bacterial infection. Upon interaction with bacteria or bacteria-derived antigens, neutrophils initiate a multi-staged response of which the terminal stage is NETosis, formation of protease-decorated nuclear DNA into extracellular traps. NETosis has a great propensity to elicit ocular damage and has been associated with diabetic retinopathy and diabetic macular oedema (DME) progression. Here, we interrogate the relationship between gut barrier dysfunction, endotoxaemia and systemic and intestinal neutrophilia in diabetic retinopathy.

**Methods:**

In a cohort of individuals with type 2 diabetes (*n*=58) with varying severity of diabetic retinopathy and DME, we characterised the abundance of circulating neutrophils by flow cytometry and markers of gut permeability and endotoxaemia by plasma ELISA. In a mouse model of type 2 diabetes, we examined the effects of diabetes on abundance and function of intestinal, blood and bone marrow neutrophils, gut barrier integrity, endotoxaemia and diabetic retinopathy severity. Pharmacological inhibition of NETosis was achieved by i.p. injection of the peptidyl arginine deiminase 4 inhibitor (PAD4i) GSK484 daily for 4 weeks between 6 and 7 months of type 2 diabetes.

**Results:**

In human participants, neutrophilia was unique to individuals with type 2 diabetes with diabetic retinopathy and DME and was accompanied by heightened circulating markers of gut permeability. At late-stage diabetes, neutrophilia and gut barrier dysfunction were seen in *db/db* mice. The *db/db* mice exhibited an increase in stem-like pre-neutrophils in the intestine and bone marrow and a decrease in haematopoietic vascular reparative cells. In the *db/db* mouse intestine, enhanced loss of gut barrier integrity was associated with elevated intestinal NETosis. Inhibition of NETosis by the PAD4i GSK484 resulted in decreased abundance of premature neutrophils in the intestine and blood and resulted in neutrophil retention in the bone marrow compared with vehicle-treated *db/db* mice. Additionally, the PAD4i decreased senescence within the gut epithelium and yielded a slowing of diabetic retinopathy progression.

**Conclusions/interpretation:**

Severity of diabetic retinopathy and DME were associated with peripheral neutrophilia, gut barrier dysfunction and endotoxaemia in the human participants. *db/db* mice exhibited intestinal neutrophilia, specifically stem-like pre-neutrophils, which was associated with elevated NETosis and decreased levels of vascular reparative cells. Chronic inhibition of NETosis in *db/db* mice reduced intestinal senescence and NETs in the retina. These changes were associated with reduced endotoxaemia and an anti-inflammatory bone marrow milieu with retention of pre-neutrophils in the bone marrow and increased gut infiltration of myeloid angiogenic cells. Collectively, PAD-4i treatment decreased gut barrier dysfunction, restoring physiological haematopoiesis and levels of haematopoietic vascular reparative cells.

**Graphical Abstract:**

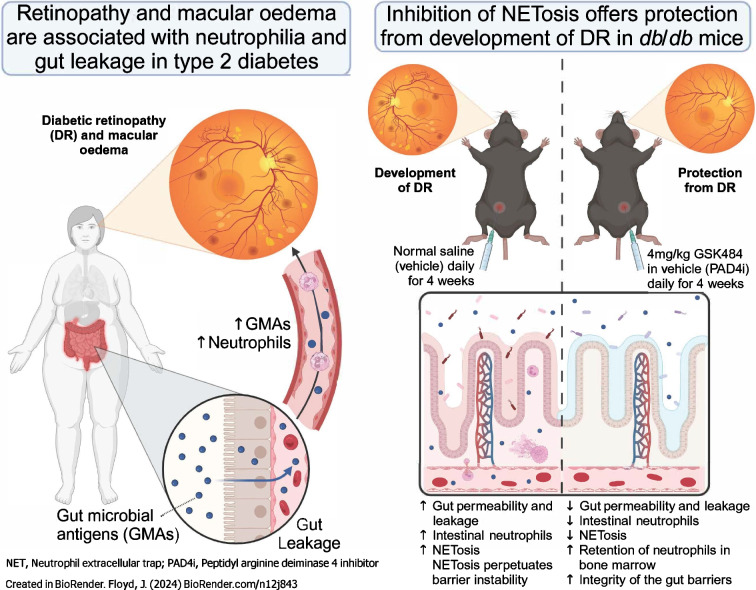

**Supplementary Information:**

The online version contains peer-reviewed but unedited supplementary material available at 10.1007/s00125-024-06349-4.


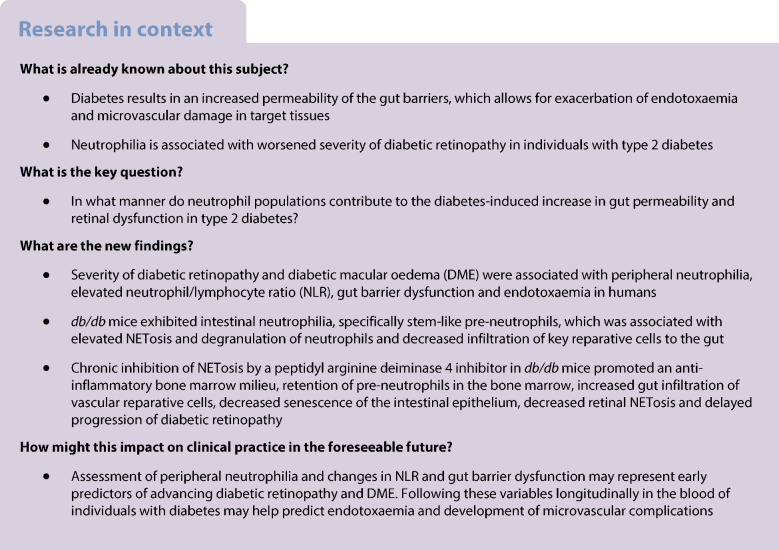


## Introduction

Type 2 diabetes is a chronic metabolic disease affecting more than 30 million individuals in the USA and hundreds of millions worldwide. The disease is characterised by hyperglycaemia, hyperinsulinaemia and dyslipidaemia [1] and is the result of progressive insulin resistance, oxidative stress and chronic low-grade inflammation that can result in development of a plethora of complications, including but not limited to diabetic retinopathy, nephropathy, neuropathy, CVD and non-alcoholic fatty liver disease [2]. The most common complication is diabetic retinopathy and affects approximately one in three diabetic individuals. Diabetic retinopathy is a disease of retinal vasodegeneration characterised by increased retinal vascular permeability, intraretinal microvascular abnormalities and microaneurysms/haemorrhages [3]. Diabetic retinopathy in humans is divided into two stages, non-proliferative diabetic retinopathy (NPDR) and proliferative diabetic retinopathy (PDR), based on the absence or presence of retinal neovascularisation, respectively [3, 4]. In addition, diabetic macular oedema (DME) develops secondary to diabetic retinopathy but occurs irrespective of diabetic retinopathy severity status and results in central vision loss. Together, diabetic retinopathy and DME are the leading causes of moderate-to-severe vision loss in people aged 25–74 years and are expected to increase in prevalence in coming years [5, 6].

The mechanisms by which diabetes induces diabetic retinopathy have been the subject of intense study over many decades and include hyperglycaemia-induced metabolic dysregulation, imbalance of the renin–angiotensin–aldosterone system and oxidative-stress-induced mitochondrial dysfunction. Aberrant inflammation, often called ‘meta inflammation’, is gut-driven systemic inflammation that provides a constant source of myeloid cells that can be recruited to the retina [7]. The role of the gut–retina axis in diabetic retinopathy pathogenesis finds support in our initial study showing that the levels of peptidoglycan and fatty acid binding protein 2 (FABP2), two serum markers of gut leakage, are increased in humans with type 1 diabetes and type 2 diabetes [8]. Subsequently, we examined the association of gut-regulated components of the immune system, gut leakage markers (FABP2 and peptidoglycan) and angiotensin II in individuals with type 1 diabetes with and without diabetic retinopathy compared with control individuals. We showed that individuals with type 1 diabetes exhibit elevations in gut-derived circulating immune cells (ILC1 cells) and higher gut leakage markers, which were positively correlated with plasma angiotensin II and diabetic retinopathy severity [9].

Diabetes-induced immune activation includes leukocyte adhesion to capillaries leading to occlusion/ischaemia, which drives the development and progression of diabetic retinopathy and DME. Heightened abundance of peripheral neutrophils and their products are associated with increased risk for development of diabetic retinopathy and DME [10–21]. An elevated neutrophil/lymphocyte ratio (NLR) has been shown to predict worse outcomes in humans who underwent DME intravitreal treatment with the vascular endothelial growth factor-A (VEGF-A) inhibitor ranibizumab. In addition to neutrophil abundance, several studies in diabetic rodents and humans have demonstrated strong associations between aberrant neutrophil function and development/progression of diabetic retinopathy and DME [11, 14, 16, 17, 22–26].

Neutrophils, the most abundant circulating leukocytes, are initial responders to bacterial infection and play a vital role in the maintenance of inflammatory homeostasis. Upon activation by bacterial ligands and proinflammatory cytokines, neutrophils have the capacity to initiate a multitude of molecular cascades aimed at recruiting additional immune cells to areas of infection, clearance of bacterial infiltrates and resolution of inflammation. These include secretion of proinflammatory cytokines/chemokines, phagocytosis of bacteria or antigen-containing extracellular fluid, release of protease-containing intracellular granules and secretory vesicles, secretion of reactive oxygen species (ROS) and neutrophil extracellular trap (NET)osis. Though these mechanisms are tightly controlled in healthy individuals, meta inflammation induces a shift within the delicate balance of the neutrophil response, potentially resulting in unintended tissue damage and chronic inflammation.

Elevation of systemic neutrophils appears unique to type 2 diabetes as this does not occur in non-diabetic individuals and individuals with type 1 diabetes [27]. Extensive human and rodent studies have demonstrated that diabetic neutrophils exhibit decreased chemotaxis, phagocytosis and apoptosis while having increased capacity for ROS secretion, proinflammatory cytokine production, and NETosis [28]. The combination of these events results in a decreased capacity for bacterial clearance and an increased propensity to induce tissue damage. In line with this, several studies have demonstrated that elevated neutrophil-derived proteases, ROS and NETs inhibit diabetic wound healing [29–34] and accelerate diabetic vascular diseases, including diabetic retinopathy [22, 35–41]. Current work by our group suggests that early intestinal pathology in type 2 diabetes induces a weakening of the epithelial and endothelial barriers within the small intestine resulting in increased translocation of gut-derived microbial antigens (GMAs) into the systemic circulation. GMAs travel to the bone marrow and act on toll-like receptors (TLRs) present on all haematopoietic populations. GMAs increase the rate of granulopoiesis and in health this is needed for proper haematopoiesis. However, due to a ‘leaky gut’ excessive GMAs enter the blood and result in exaggerated granulopoiesis. We have shown that in the retina, increased levels of circulating GMAs activate non-canonical TLR2-mediated signalling in retinal endothelial cells to promote vascular leakage and disruption of the blood–retina barrier, initiating and perpetuating diabetic retinopathy [8]. By modulating gut barrier integrity, we have demonstrated the ability to reverse or prevent the development of diabetic retinopathy in a mouse model of type 1 diabetes [8, 42]; however, type 1 diabetes is not characterised by excessive gut inflammation yet leakage of GMAs exist, in particular peptidoglycans [8, 42]. We showed the critical role of myeloid angiogenic cells (MACs) by repleting them in the type 1 diabetes gut using adoptive transfer [8].

Taken together, there is a paucity of understanding of gut inflammation in diabetes and unique differences between type 1 and type 2 that cannot be attributed to hyperglycaemia. The role of intestinal neutrophils has yet to be studied in the context of type 2 diabetes and diabetic retinopathy. In fact, the influence of intestinal neutrophils on gut barrier dysfunction even outside of the diabetic context is unknown. In this study, we postulated that hyperactivated intestinal neutrophils participate in the breakdown of the gut barrier either directly by the cytotoxic effects of their bactericidal functions or by interfering with the recruitment of MACs inhibiting vascular repair mechanisms. We aimed to determine the extent to which an aberrant intestinal neutrophil response may play a role in the exacerbation of gut barrier dysfunction and development of meta inflammation and how it may contribute to diabetic retinopathy. To determine the role of NETosis in this process, we performed pharmacological inhibition of NETosis with the peptidyl arginine deiminase 4 inhibitor (PAD4i) GSK484 in *db/db* mice with 6 months of type 2 diabetes. This work is the first to interrogate the intestinal neutrophil compartment in type 2 diabetes and lends valuable support for the role of aberrant intestinal homeostasis in the pathogenesis of type 2 diabetes, diabetic retinopathy and other diabetic vascular complications.

## Methods

### Human participants

Blood was collected at the UAB Callahan Eye Hospital and Clinics according to Institutional Review Board (IRB) guidelines and under the approval of IRB protocols 300000173, 300000068 and 300000188. Whole blood was collected from individuals with type 2 diabetes with varying severities of diabetic retinopathy and DME. Diabetic retinopathy severity was assessed by fellowship trained retinal specialists via fundus examination and graded according to the diabetic retinopathy international classification system [43]. Presence or absence of DME was determined by spectrum domain optical coherence tomography. All blood was placed into EDTA-coated plasma tubes and non-EDTA-coated serum tubes. All blood collections occurred during the morning hours to reduce possible circadian-related variations in immune and metabolic outcomes. Plasma and serum samples were stored at −80°C until processed. A total of 59 healthy control individuals, 27 individuals with type 2 diabetes without eye disease, and 31 individuals with type 2 diabetes with diabetic retinopathy ± DME were assessed for abundance of peripheral immune cells, markers of metabolic dysfunction and markers of gut permeability/endotoxaemia. Demographic characteristics, relevant biological variables, clinical interventions and individual stratification by cohort are included in Table 1. See electronic supplementary material (ESM) Methods for further details.

**Table 1:** Demographic and clinical characteristics of human participants

Variable	Healthy control individuals	Individuals with type 2 diabetes				
		Without DME, without diabetic retinopathy	Without DME, with NPDR	Without DME, with PDR	With DME, with NPDR	With DME, with PDR
No. of individuals	59	27	4	6	12	9
Sex						
Male	27 (46)	12 (44)	2 (50)	1 (17)	6 (50)	5 (56)
Female	32 (54)	15 (56)	2 (50)	5 (83)	6 (50)	4 (44)
Age range						
<30 years	9 (15)	0	0	0	0	0
30–50 years	22 (37)	6 (22)	1 (25)	2 (33)	2 (17)	5 (56)
>50 years	27 (46)	21 (78)	3 (75)	4 (67)	10 (83)	4 (44)
Unknown	1 (2)	0	0	0	0	0
Diabetes duration						
<10 years	-	1 (4)	0	0	0	0
10–20 years	-	4 (15)	0	1 (17)	2 (17)	0
20–30 years	-	6 (22)	1 (25)	3 (50)	3 (25)	4 (44)
30–40 years	-	4 (15)	1 (25)	1 (17)	0	0
>40 years	-	0	0	1 (17)	0	1 (11)
Unknown	-	12 (44)	2 (50)	0	7 (58)	4 (44)
Race						
African American	18 (31)	11 (41)	0	2 (33)	9 (75)	2 (22)
White	38 (64)	15 (56)	4 (100)	4 (67)	3 (25)	7 (78)
Other	3 (5)	1 (4)	0	0	0	0
Blood glucose						
<5.55 mmol/l	23 (39)	6 (22)	0	1 (17)	3 (25)	0
5.55–7.21 mmol/l	7 (12)	3 (11)	2 (50)	0	1 (8)	0
>7.21 mmol/l	10 (17)	12 (44)	0	4 (67)	6 (50)	1 (11)
Unknown	19 (32)	6 (22)	2 (50)	1 (17)	2 (17)	8 (89)
HbA_**1c**_						
<39 mmol/mol (<5.7%)	27 (46)	5 (19)	0	0	0	0
39–46 mmol/mol (5.7–6.4%)	3 (5)	3 (11)	0	0	2 (17)	1 (11)
>46 mmol/mol (>6.4%)	0	7 (26)	2 (50)	1 (17)	5 (42)	1 (11)
Unknown	29 (49)	12 (44)	2 (50)	5 (83)	5 (42)	7 (78)
Complications						
CVD	0	0	0	0	0	0
Nephropathy	0	0	0	0	0	0
Neuropathy	0	1 (4)	0	0	0	0
Medication(s)						
Cholesterol-lowering	1 (2)	21 (78)	4 (10)	3 (50)	10 (83)	5 (56)
Glucose-lowering	2 (3)	27 (100)	4 (100)	6 (100)	6 (50)	5 (56)
Antihypertensive	3 (5)	11 (41)	3 (75)	3 (50)	5 (42)	2 (22)
Antineuropathic	0	1 ( 4)	0	0	0	0

Data are shown as *n* (%)

### Human peripheral blood flow cytometry

Whole blood was collected in EDTA-coated plasma tubes. For each flow cytometry panel, 200 µl of whole blood was lysed (ACK RBC Lysis Buffer catalogue no. A1049201, Thermo Fisher Scientific, Waltham, MA, USA) and circulating cell pellets were isolated, stained and acquired under standard procedures. Detailed protocols for preparation of samples for flow cytometry, acquisition and compensations are included in ESM, antibodies used in flow cytometry studies can be found in ESM Table 1.

### Housing, metabolic evaluation and genotyping of experimental mice

Founder breeding pairs of Akita (C57BL/6-*Ins2*^Akita^/J, Jax strain no. 003548) and *db/db* (B6.BKS(D)-*Lepr*^db^/J, Jax strain no. 000697) mice were purchased from The Jackson Laboratory. The experimental protocol for the animal studies (Animal protocol numbers : 21196 and 20919) were approved by the Institutional Animal Care and Use Committee (IACUC) at University of Alabama at Birmingham. Metabolic characteristics and genotyping of Akita and *db/db* mice have been previously published [8]. Identification of gene status in heterozygous Akita and homozygous *db/db* mice was confirmed by genotyping using RT-PCR followed by agarose gel electrophoresis. For genotyping primer sequences, see ESM Table 2. See ESM Methods for further details. At 2 months of age, after diabetes development was confirmed, the mice were randomly assigned to diabetic cohorts for each time point of the study. Only male mice were used in the studies.

## Mouse blood and tissue isolation

Mice were anaesthetised with isoflurane prior to cervical dislocation. Plasma was obtained and stored at −80°C until analysis. From each mouse, femurs and tibias were isolated and used in flow cytometric studies, RNA and protein studies, and immunofluorescence studies. For assessment of ocular pathology, one eye fixed in 4% wt/vol. paraformaldehyde (PFA) was used for staining of acellular capillaries and the other eye was prepared as frozen tissue sections for immunohistochemistry. From each mouse, approximately 4 cm of distal ileum was flushed and divided into pieces for protein and RNA studies, immunofluorescence studies, and flow cytometric studies.

### Murine flow cytometry studies

Whole blood was collected in EDTA-coated tubes. For the peripheral blood flow cytometry panel, 200 µl of whole blood was lysed (ACK RBC Lysis Buffer catalogue no. A1049201, Thermo Fisher Scientific) and circulating cell pellets were isolated, stained and acquired under standard procedures. For bone marrow studies, bones were cut at one end and placed into a 0.5 ml Eppendorf tube, which was punctured at the bottom with a 21G needle. Each 0.5 ml tube was placed within a 1.5 ml Eppendorf tube containing 200 μl FACS buffer and centrifuged at 1610 *g* for 1 min at 4°C to remove the marrow. Marrow was resuspended in 1 ml RBC lysis buffer and incubated for 5 min on ice. Cells were washed with 1 ml FACS buffer and stained for flow cytometry. For small intestine studies, ileum sections were flushed of faecal contents and split longitudinally to expose luminal cells. Tissue underwent epithelial stripping followed by digestion in collagenase buffer. Tissue suspensions were filtered through a 40 μm nylon filter and centrifuged at 300 *g* for 5 min at 4°C to pellet cells. Cells were washed in FACS buffer before undergoing staining for flow cytometry. For antibodies used in flow cytometry studies, see ESM Table 1 and for gating strategies, see ESM Fig. 1.

### Immunofluorescence staining of murine tissues

Ileum sections were flushed of luminal contents with chilled PBS before undergoing fixation in 4% PFA (wt/vol.) for 2 h (ileum) or 30 min (eyes) at 4°C. Tissue was washed with PBS before undergoing dehydration in 15% and 30% sucrose in 1× PBS, each for 24 h, at 4°C. Dehydrated tissue was then embedded in either paraffin or Tissue-Tek Optimal cutting temperature (OCT) compound (Sakura Finetek USA) before sectioning. Paraffin sections underwent paraffin removal and rehydration along classic procedures before undergoing antigen retrieval, neutralisation of endogenous peroxidases, permeabilisation, blocking and staining with primary and secondary antibodies. Cryosections were thawed at room temperature for 30 min then washed with PBS. Sections were permeabilised, blocked and stained with primary and secondary antibodies. Images were collected with a Nikon AX-R confocal microscope and analysed with NIS-Elements v5.42 software (Nikon Instruments, Melville, NY 11747-3064, USA). The experimenters were masked to group assignments during outcome assessment.

For antibodies used in immunofluorescence studies, see ESM Table 3. See ESM Methods for further details.

### ELISAs for markers of gut permeability and endotoxaemia

Plasma concentrations of proteins of interest were detected in human and murine plasma using ELISA kits following the manufacturers’ instructions. For details of ELISA kits, see ESM Table 4. The experimenters were masked to group assignments during outcome assessment.

### Western blot

Tissue lysates (cytoplasmic and nuclear fractions) were prepared from flash-frozen small intestine (jejunum and ileum) from all cohorts using a Cell Fractionation Kit purchased from Cell Signaling Technology (Boston, MA, USA, no. 9038) following the manufacturer’s instructions and containing protease inhibitor cocktail. Protein concentrations were measured using a colorimetric BCA protein assay. Equal concentrations of protein were resolved using SDS-PAGE gels and transferred onto nitrocellulose membranes. Membranes were blocked and incubated with primary antibody overnight at 4°C. Blots were washed and incubated with secondary antibody for 2 h at room temperature followed by washing. Protein bands were visualised using chemiluminescence reagents followed by stripping and reprobing with β-actin antibody to confirm equal protein loading. For antibodies used in western blot studies, see ESM Table 5. The experimenters were masked to group assignments during outcome assessment.

### Murine neural retina flat-mount preparation, immunofluorescence and acellular capillaries

Retinas were isolated from whole globes and cleaned. For acellular capillaries, flat mounts were digested in elastase for 1.5–2 h to remove the internal limiting membrane followed by incubation in 0.5% Triton-X100 to remove the neural retina. Retinas were flat-mounted onto glass slides and underwent counterstaining with haematoxylin before being imaged. For vascular endothelial (VE)-cadherin staining, retinal flat mounts were permeabilised with 0.1% Triton-X100 for 15 min before undergoing blocking and staining. Detailed protocols and recipes for preparation and immunostaining of the retinas for VE-cadherin and for elastase digestion for acellular capillaries are described in ESM Methods. The experimenters were masked to group assignments during outcome assessment.

### Peptidyl arginine deiminase 4 inhibition by GSK484

Inhibition of NETosis was performed via administration of the selective, reversible PAD4i GSK484 (MedChemExpress HY-100514). Male *db/db* mice were randomly divided into two groups (*n*=5 each), which received daily i.p. injections of either saline vehicle (200 µl, 0.9% wt/vol. normal saline [154 mmol/l NaCl]) or GSK484 (4 mg/kg in 200 µl normal saline) over 4 weeks between 6 and 7 months of diabetes duration in male *db/db* mice. Non-injected wild-type (WT) mice (*n*=5) served as controls. No adverse events were noted. The experimenters were masked to group assignments during outcome assessment.

### Data analysis and statistics

All analyses were performed in GraphPad Prism v9.1 software (https://www.graphpad.com/). Prior to hypothesis testing, data were assessed for outliers, as indicated by the ROUT method, and outliers were excluded from further analyses. Data underwent assessment for adherence to a normal (Gaussian) distribution using the Shapiro–Wilks normality test. Data that were found to be normally distributed were analysed by either an unpaired Student’s *t* test (categorical predictor with two levels) or a one-way ANOVA (categorical predictor with more than two levels). Parametric one-way ANOVA results were adjusted to account for multiple comparisons using Tukey’s HSD procedure. Data that did not meet the assumptions of normality were analysed by either an unpaired Mann–Whitney test (Wilcoxon rank sum test, categorical predictor with two levels) or a Kruskal–Wallis one-way ANOVA (categorical predictor with more than two levels). Non-parametric Kruskal–Wallis one-way ANOVA results were adjusted to account for multiple comparisons using Dunn’s procedure. For all statistical analyses, each input measure is indicative of a single, independent sample from one human participant or one experimental animal. All data were analysed at α=0.05 unless explicitly stated otherwise. All *p* values presented in figures/tables, except those of *t* tests, are adjusted for multiple comparisons.

## Results

### Individuals with type 2 diabetes with diabetic retinopathy and DME exhibit peripheral neutrophilia, increased gut permeability and endotoxaemia

**Fig. 1:**
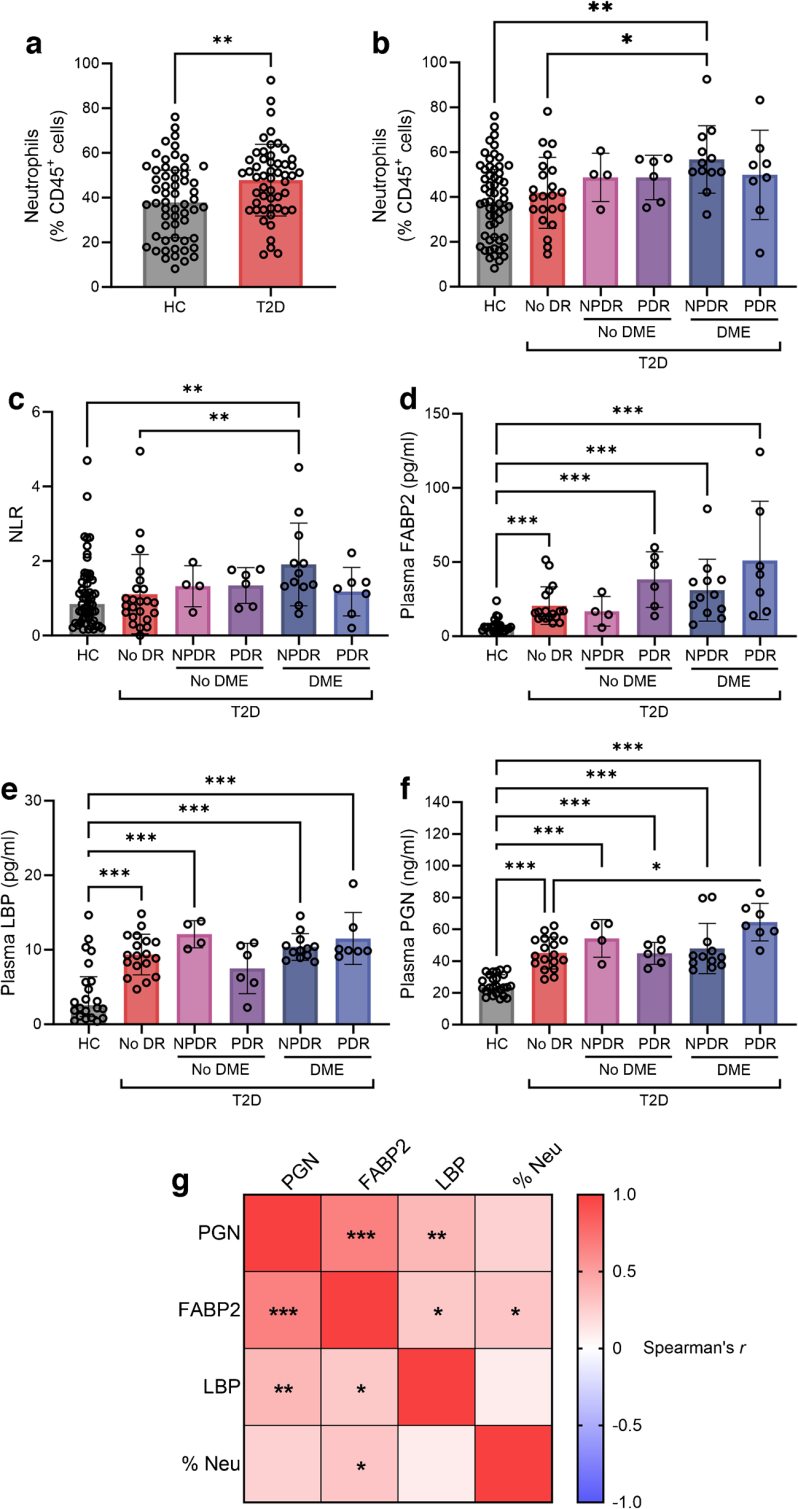
Individuals with type 2 diabetes with diabetic retinopathy and DME exhibit peripheral neutrophilia and elevated NLR associated with elevated circulatory markers of gut permeability and endotoxaemia. In a cohort of individuals with type 2 diabetes with varying severity of diabetic retinopathy/DME and age- and sex-matched healthy control individuals, we assessed the abundance of circulating neutrophils (CD45^+^CD15^+^SSC^hi^) by flow cytometry. (**a**) Quantification of neutrophil abundance in individuals with type 2 diabetes, irrespective of eye disease severity, compared with healthy control individuals. (**b**, **c**) Quantification of neutrophils (**b**) and NLR (**c**) in individuals with type 2 diabetes stratified by severity of eye disease. (**d**–**f**) Quantification of FABP2 (**d**), LBP (**e**) and PGN concentrations (**f**) as determined by ELISA. (**g**) Bivariate correlations of human neutrophil abundance and plasma FABP2, LBP and PGN. Each data point is indicative of one independent measure from one participant (HC, *n*=59; T2D_No DR_, *n*=27; T2D_NPDR without DME,_ *n*=4; T2D_PDR without DME,_ *n*=6; T2D_NPDR with DME,_ *n*=12; T2D_PDR with DME,_ *n*=9). *p<0.05, **p<0.01, ***p<0.001. DR, diabetic retinopathy; HC, healthy control; Neu, neutrophils; T2D, type 2 diabetes

A total of 58 individuals with established type 2 diabetes and varying severities of diabetic retinopathy and DME, along with age- and sex-matched healthy control individuals were enrolled in this study. Participant demographic and clinical characteristics are detailed in Table 1. To assess associations between diabetic eye disease and neutrophilia, we performed flow cytometry of peripheral blood. Peripheral neutrophil abundance (CD45^+^CD15^+^SSC^hi^) was confirmed to be significantly elevated in individuals with type 2 diabetes, irrespective of eye pathology, compared with control individuals (Fig. 1a). Upon stratification by severity of diabetic retinopathy and presence of DME, we observed significantly elevated circulating neutrophils (Fig. 1b) and NLR (Fig. 1c) in individuals with NPDR with DME compared with individuals with type 2 diabetes without eye disease and healthy individuals. These data confirm previous observations of neutrophilia and elevated NLR in diabetic individuals that are exacerbated in diabetic individuals with severe eye disease [25, 44–49].

To determine whether neutrophilia is associated with increased permeability of the gut barrier and translocation of GMAs into the systemic circulation, we examined plasma markers of gut permeability (FABP2) and endotoxaemia (lipopolysaccharide-binding protein [LBP] and peptidoglycan [PGN]) by ELISA. We previously showed that FABP2 and PGN were elevated in the plasma of individuals with type 1 diabetes [8, 42]. Compared with control individuals, FABP2 levels were significantly increased in individuals with type 2 diabetes, with the exception of those with NPDR and no DME (Fig. 1d). LBP was found to be significantly elevated in all type 2 diabetes groups except for individuals with PDR and no DME compared with control individuals (Fig. 1e). PGN was found to be significantly elevated in all type 2 diabetes groups compared with control individuals. However, only individuals with PDR and DME exhibited heightened PGN compared with individuals with type 2 diabetes without eye disease (Fig. 1f). Using Spearman’s correlation analysis, we found statistically significant positive correlations between PGN vs FABP2 (*r*=0.66, *p*=3.84 × 10^−10^), PGN vs LBP (*r*=0.37, *p*=0.00137) and FABP2 vs LBP (*r*=0.29, *p*=0.0156). Peripheral neutrophil abundance was only significantly correlated with FABP2 (*r*=0.30, *p*=0.014), a marker specific for gut epithelial injury (Fig. 1g). Together, these data suggest that loss of epithelial gut barrier integrity, as demonstrated by increased plasma FABP2, contributes to the presence of GMAs in the circulation and the skewing of haematopoiesis towards generation of neutrophils. We next asked whether extravasated circulating neutrophils could exacerbate gut barrier damage, suggesting ‘dual directionality’ wherein intestinal neutrophilia promotes further gut damage to initiate and perpetuate ocular pathology in type 2 diabetes.

### *db/db* mice but not Akita mice exhibit intestinal neutrophilia throughout diabetes duration

**Fig. 2:**
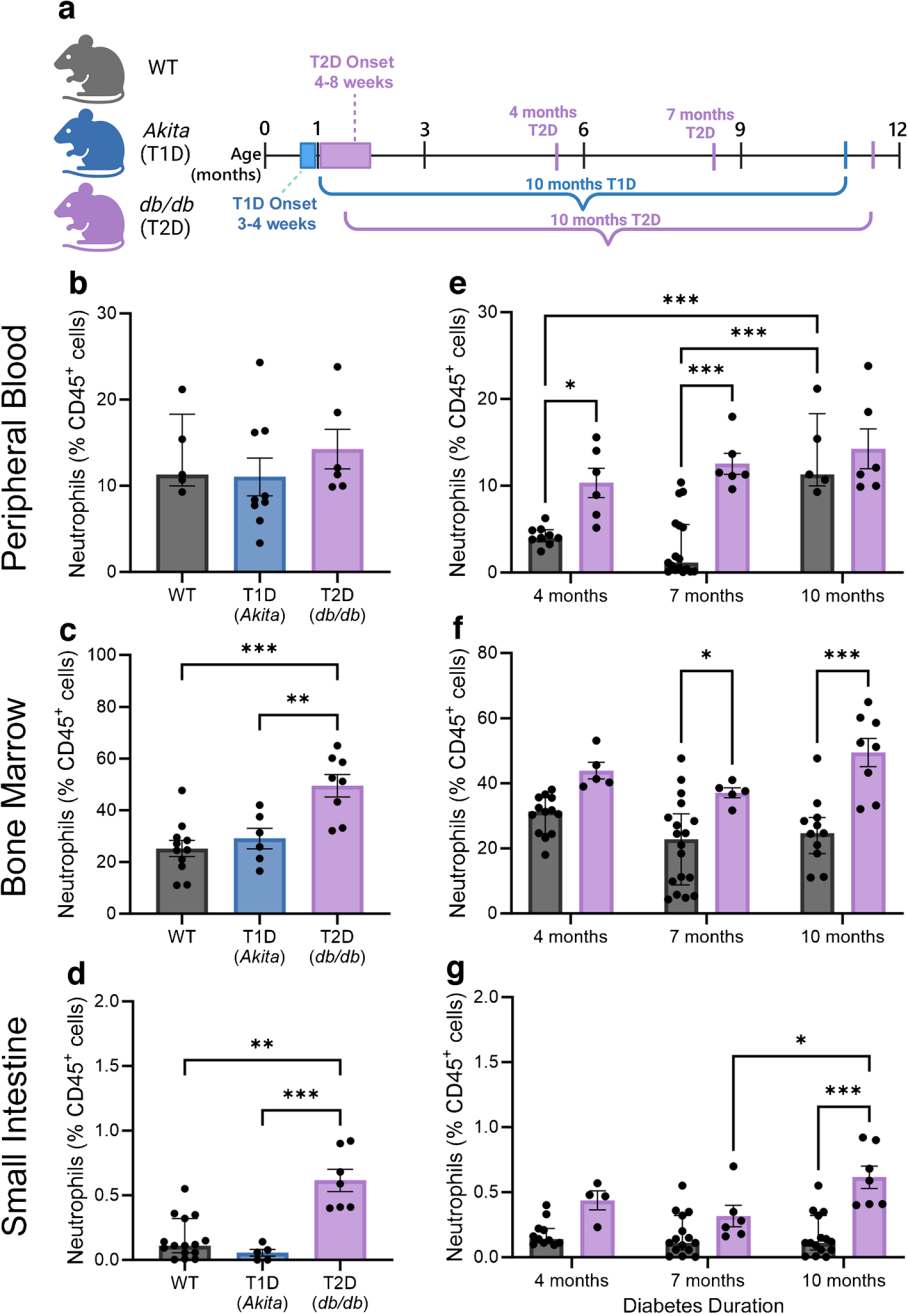
Expansion of the central and small intestine neutrophil compartments occurs in mice with type 2 diabetes but not in those with type 1 diabetes. (**a**) Schematic detailing timeline of studies comparing the mouse models of type 1 diabetes (Akita) and type 2 diabetes (*db/db*) with 10 months of respective diabetes duration along with secondary and tertiary cohorts of *db/db* mice euthanised at 4 and 7 months of type 2 diabetes duration. We used flow cytometry to assess the abundance of neutrophils (CD45^+^CD11b^+^Ly6G^+^) in intestinal and central compartments. (**b**–**d**) Quantification of neutrophil abundance in the peripheral blood (**b**), bone marrow (**c**) and small intestine (**d**) of Akita (type 1 diabetes) and *db/db* (type 2 diabetes) mice with 10 months of respective diabetes duration and age-matched WT mice. (**e**–**g**) Quantification of neutrophil abundance in the peripheral blood (**e**), bone marrow (**f**) and small intestine (**g**) of *db/db* (type 2 diabetes) mice with 4, 7 and 10 months of diabetes duration and age-matched WT mice. Each data point represents one independent observation from one experimental animal (*n*=4–18 per group). *p<0.05, **p<0.01, ***p<0.001. T1D, type 1 diabetes; T2D, type 2 diabetes

To understand the molecular mechanism responsible for the observed systemic neutrophilia and to elucidate the influence, if any, of neutrophilia on the development of gut barrier dysfunction and endotoxaemia, we transitioned to the use of mouse models of diabetes. We reassessed the Akita mouse model of type 1 diabetes [8] for comparison with the *db/db* mouse model of type 2 diabetes at 10 months of diabetes duration (timeline detailed in Fig. 2a). At 10 months of diabetes, both Akita and *db/db* mice exhibit diabetic retinopathy [50, 51]. At 10 months of diabetes, we observed a non-significant increase in the proportion of neutrophils within the peripheral blood of *db/db* mice (Fig. 2b). A significant increase in abundance of bone marrow neutrophils was observed in *db/db* mice compared with Akita and WT mice (Fig. 2c). A significant increase in neutrophil abundance was also observed in the small intestine (Fig. 2d). Importantly, no difference in neutrophil abundance was observed at these sites when comparing WT and Akita mice. These data suggest that neutrophilia is a phenotype exclusive to rodent models of type 2 diabetes and that the intestine may be a site of heightened neutrophil activity. We next examined whether neutrophilia occurred in the initial stages of type 2 diabetes or occurred only in the later stages of the disease.

In complementary experiments to those in aged mice, we studied cohorts of *db/db* mice with 4 and 7 months of type 2 diabetes duration. Increased peripheral blood neutrophil abundance was observed at 4 and 7 months but the increase did not reach statistical significance at 10 months in *db/db* mice compared with WT mice (Fig. 2e). In the bone marrow of *db/db* mice, we observed a non-significant increase in neutrophils at 4 months and significantly elevated neutrophils at 7 and 10 months of type 2 diabetes compared with WT mice (Fig. 2f). Within the small intestine of *db/db* mice, we observed non-significant increases in neutrophils at 4 and 7 months and significantly elevated neutrophil abundance at 10 months of type 2 diabetes when compared with WT mice; significantly increased intestinal neutrophil abundance was observed in *db/db* mice with 10 months of diabetes duration compared with those with 7 months of diabetes duration (Fig. 2g). Together, these rodent data support previous data from studies in humans, which had established systemic neutrophilia as a characteristic of type 2 diabetes [52–54]. Our studies extend these findings to the small intestine where neutrophilia is a persistent phenomenon throughout the time course of diabetes we studied.

We postulated that intestinal neutrophilia may play a regulatory role in gut barrier integrity in type 2 diabetes. Gut barrier dysfunction drives endotoxaemia, which is a critical activator of systemic inflammation; thus, increased numbers of neutrophils could impair barrier integrity rather than promote resolution of inflammation. A key function of neutrophils is the formation of neutrophil extracellular traps (NETosis), the expulsion of protease-decorated nuclear DNA into the extracellular space to ‘trap’ perceived pathogens. We next investigated whether intestinal neutrophilia was associated with heightened NETosis.

### Intestinal NETosis is heightened in *db/db* mice

**Fig. 3:**
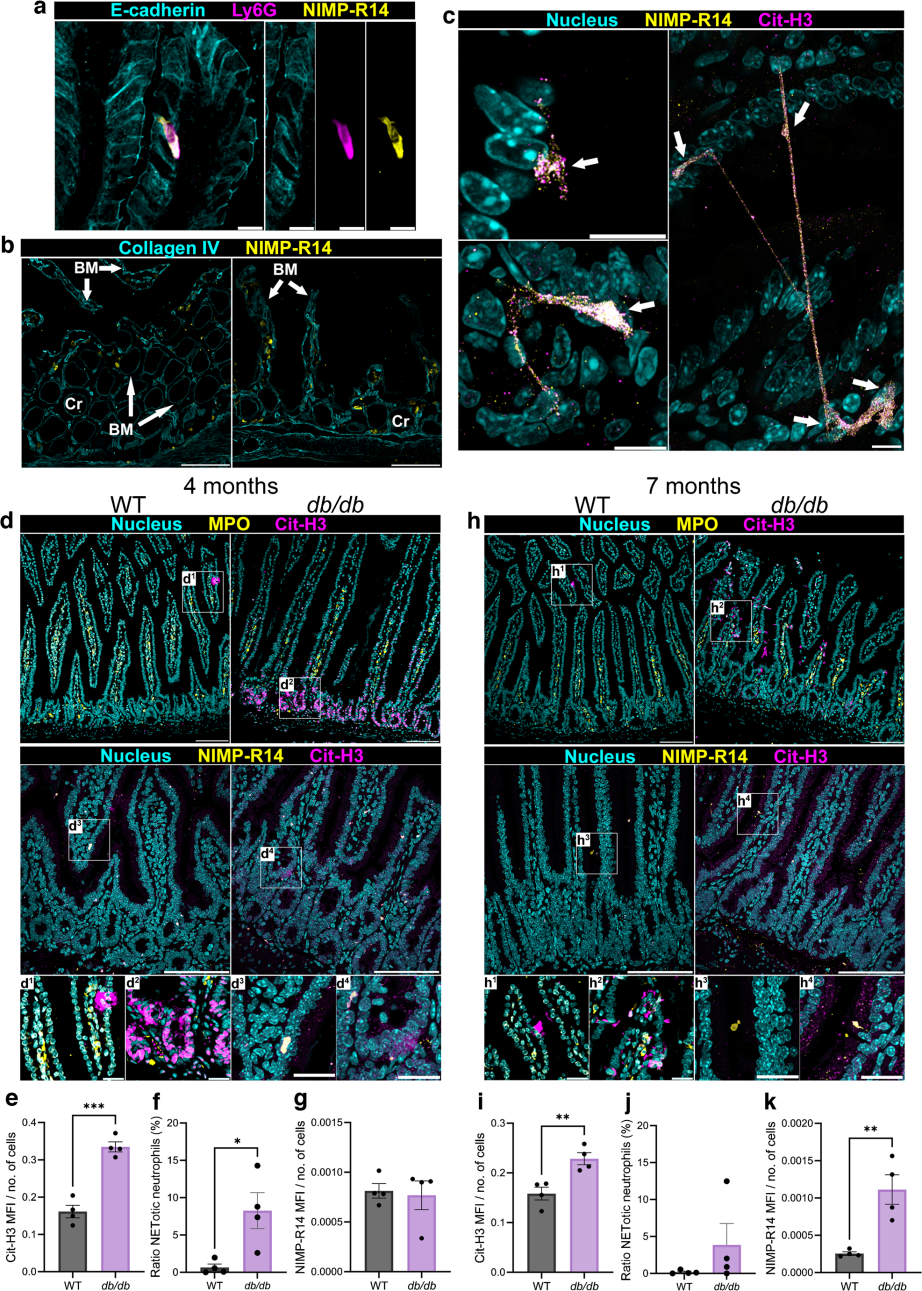
*db/db* mice exhibit heightened intestinal NETosis throughout diabetes duration. To assess whether mice with type 2 diabetes mice experience elevated NETosis in the small intestine, we performed confocal IF microscopy of FFPE tissue sections from *db/db* mice with 4 and 7 months duration of diabetes. We identified intestinal neutrophils as being Ly6G^+^NIMP-R14^+^ cells (**a**). We found neutrophils (yellow) localised in the intra-epithelial space (**a**; scale bar, 10 µm) and between epithelial and endothelial basement membranes, and between intestinal crypts as indicated by collagen IV staining (**b**; scale bar, 100 µm). Variations in NET structure were demonstrated and found to be comprised of one or more cells, each white arrow indicates a cell body (**c**; scale bar, 10 µm). (**d**–**g**) Representative images of Cit-H3 co-staining with the neutrophil markers MPO and NIMP-R14 at 4 months are shown (**d**; scale bar, 100 μm [low power] and 25 μm [high power]), together with quantification of Cit-H3 MFI per total cells (**e**), ratio of NETotic neutrophils (CitH3^+^MPO^+^ cells / CitH3^−^MPO^+^ cells) (**f**) and NIMP-R14 MFI per total cells (**g**). (**h**–**k**) Representative images of Cit-H3 co-staining with the neutrophil markers MPO and NIMP-R14 at 7 months are shown (**h**; scale bar, 100 μm [low power] and 25 μm [high power]), together with quantification of Cit-H3 MFI per total cells (**i**), ratio of NETotic neutrophils (CitH3^+^MPO^+^ cells / CitH3^−^MPO^+^ cells) (**j**) and NIMP-R14 MFI per total cells (**k**). Each data point represents the average of three or four randomly dispersed images taken from the tissue of one experimental animal (*n*=4 mice per group). *p<0.05, **p<0.01, ***p<0.001. BM, basement membrane; Cr, crypt

To assess whether *db/db* mice exhibit elevated intestinal NETosis, we performed confocal immunofluorescence (IF) microscopy of formalin-fixed paraffin-embedded (FFPE) small intestine tissue sections of *db/db* mice with 4 and 7 months of type 2 diabetes. To aid visualisation of gut neutrophils, we used the common neutrophil marker NIMP-R14 and demonstrated its colocalisation with the neutrophil-specific marker Ly6-G within the epithelial (E)-cadherin-expressing intestinal epithelium (Fig. 3a). Prior to activation, neutrophils surveil the mucosal and submucosal parenchyma for bacterial antigens. Using an antibody against collagen IV, we demonstrate neutrophils within the intestinal basement membrane, which facilitates stability of the endothelium (Fig. 3b). We observed NETs with a variety of morphologies. Neutrophils that had not undergone NETosis were visualised as whole cells (yellow, Fig. 3c) with punctate intracellular citrullinated histone 3 (Cit-H3) staining (magenta, Fig. 3c). Upon NETosis, the plasma membrane of neutrophils is ruptured and intracellular contents are expelled into the extracellular space. Depending on the location of the neutrophils’ bacterial ‘target’, NETs can take a multitude of forms including those that are closely localised to the neutrophil’s location or long strands that can reach to adjacent villi (Fig. 3c). One or more neutrophil(s) can participate in a single NETotic event (cell bodies are indicated by arrows in Fig. 3c).

To determine whether *db/db* mice exhibit increased intestinal NETosis compared with WT mice, we performed IF staining using the NET marker Cit-H3. Cit-H3 was colocalised with myeloperoxidase (MPO) and NIMP-R14 in *db/db* mice with 4 (Fig. 3d–g) and 7 months (Fig. 3h–k) of diabetes duration. At 4 months of type 2 diabetes, *db/db* mice exhibited significantly increased intestinal expression (mean fluorescence intensity [MFI]) of Cit-H3 normalised to the number of DAPI^+^ nuclei (Fig. 3e). To assess the degree of NETosis in the gut, we determined the ratio of intestinal neutrophils undergoing NETosis to total neutrophils (CitH3^+^MPO^+^DAPI^+^ cells / CitH3^−^MPO^+^DAPI^+^ cells). We found that the proportion of NETotic neutrophils was significantly elevated in *db/db* mice with 4 months of diabetes duration (Fig. 3f). However, elevated expression of NIMP-R14 was not observed at 4 months of diabetes duration (Fig. 3g). After 7 months of type 2 diabetes, *db/db* mice exhibited elevated intestinal Cit-H3 expression (Fig. 3i), though the increase in the proportion of NETotic neutrophils did not reach statistical significance (Fig. 3j). A significant elevated expression of NIMP-R14 was observed in the intestine of *db/db* mice with 7 months of diabetes duration when compared with WT mice (Fig. 3k). Together, these data support the notion that the gut in type 2 diabetes is a site of enhanced neutrophil activity, particularly the effector function of NETosis. Given that NETs are formed of a multitude of proteases and ROS, heightened NETosis may induce weakening of the gut epithelial and endothelial barriers and prevent their repair by vascular reparative cells.

### *db/db* mice exhibit elevated gut permeability in association with heightened NETosis

To assess whether the time course of NET detection is associated with disruption of the gut barrier, we performed immunofluorescence experiments to visualise epithelial and endothelial cell junction proteins. Epithelial junction stability was assessed by expression of the tight junction protein zonula occludens 1 (ZO-1) and the adherens junction proteins p120-catenin and epithelial cadherin (E-cadherin). Endothelial integrity was assessed by visualisation of VE-cadherin. We found the expression of the epithelial markers ZO-1, p120-catenin, and E-cadherin to be significantly decreased in *db/db* mice with 4 months of diabetes duration (ESM Fig. 2a–e). The expression of VE-cadherin was observed to be significantly elevated in the small intestine of *db/db* mice with 4 months of diabetes duration (ESM Fig. 2f, g). At 7 months of diabetes duration, we observed no difference in the expression of ZO-1 and p120-catenin but did find significantly increased E-Cadherin expression (ESM Fig. 2h–l). The expression of VE-cadherin was observed to be significantly decreased in the small intestine of *db/db* mice with 7 months of diabetes duration (ESM Fig. 2m, n). This unexpected data indicates that the integrity of the gut epithelial and endothelial barriers appears to fluctuate during the time course of diabetes. We interpreted this as a possible compensatory response of one barrier for the other. Interestingly, the NET markers were more abundant at both time points in the diabetic mice compared with the controls, not supporting the notion that one cell population (endothelium vs epithelium) is more susceptible to the influence of NETosis.

**Fig. 4:**
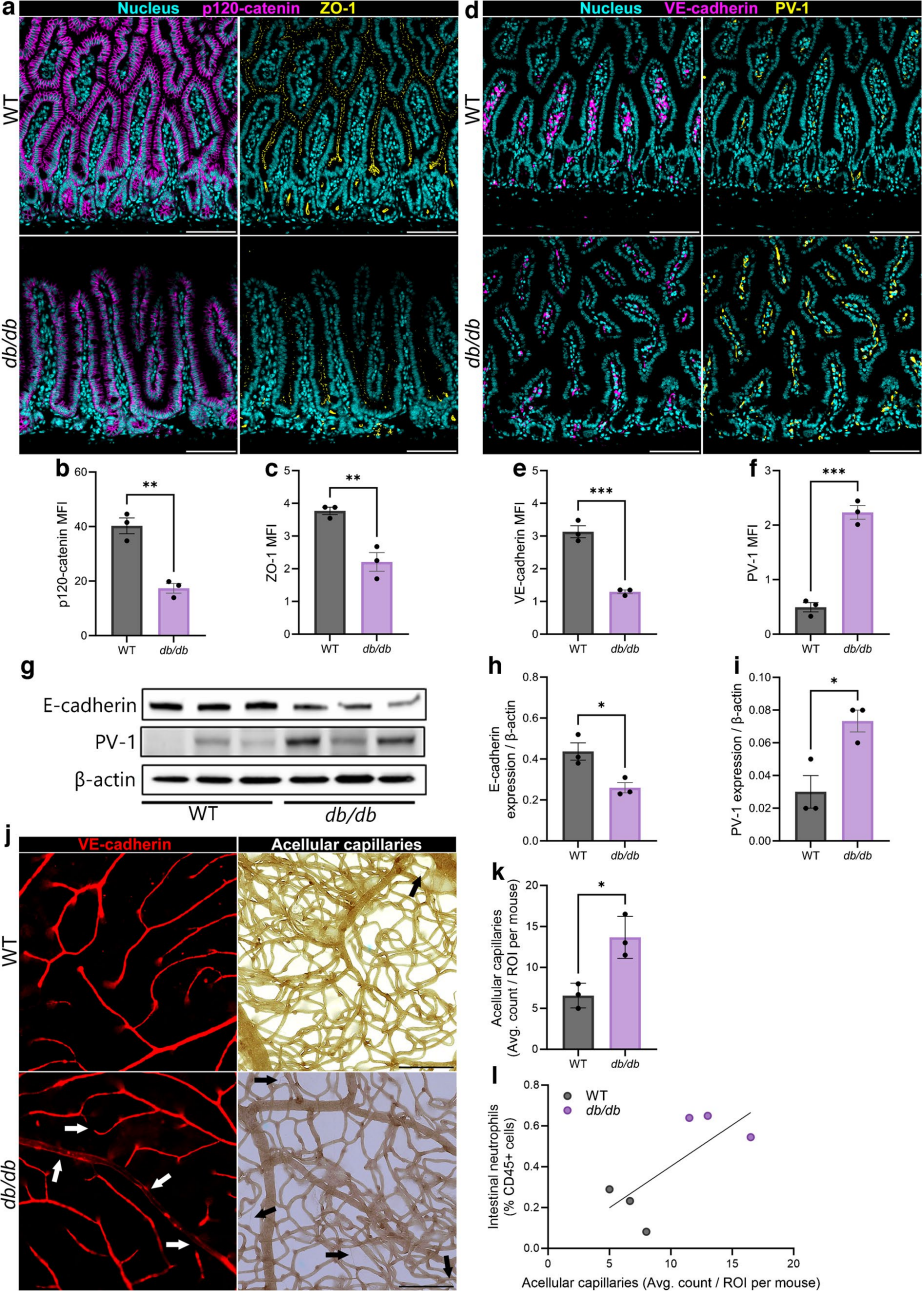
*db/db* mice exhibit loss of gut barrier integrity at 10 months of type 2 diabetes. Using confocal microscopy and western blot, we assessed the expression of epithelial- and endothelial-specific gut barrier junction proteins that are known to decrease (p120-catenin, ZO-1 and VE-cadherin) or increase (PV-1) with gut barrier permeability. (**a**–**c**) Representative images (**a**) of epithelial p120-catenin and ZO-1 staining, together with quantification of p120-catenin (**b**) and ZO-1 (**c**) MFI. (**d**–**f**) Representative images of endothelial VE-cadherin and PV-1 staining (**d**), together with quantification of VE-cadherin (**e**) and PV-1 (**f**) MFI. (**g**–**i**) Images of western blots indicating expression of E-cadherin, PV-1 and β-actin (**g**), and quantification of E-cadherin (**h**) and PV-1 (**i**) expression, each normalised to β-actin loading control. (**j**) Representative images of retinal VE-cadherin and acellular capillary staining. In VE-cadherin images, white arrows indicate gaps in staining. In acellular capillary images, black arrows indicate individual acellular capillaries. (**k**) Enumeration of retinal acellular capillaries. (**l**) Correlation between intestinal neutrophil abundance and number of acellular capillaries. In IF experiments, each data point represents the average of three randomly dispersed images taken from the tissue of one experimental mouse (*n*=3 mice per group). In western blot analysis, each data point represents one independent observation from one experimental mouse (*n*=3 mice per group). *p<0.05, **p<0.01, ***p<0.001. Scale bars, 100 µm. ROI, region of interest

To further examine barrier integrity in the *db/db* mouse model, we performed similar experiments in tissue of *db/db* mice with 10 months of diabetes duration. We found the expression of p120-catenin and ZO-1 to be significantly decreased in *db/db* mice with 10 months of diabetes duration compared with WT mice (Fig. 4a–c). We also examined the expression of VE-cadherin and found it to be reduced in *db/db* mice (Fig. 4d, e). We previously demonstrated that plasmalemma vesicle-associated protein 1 (PV-1) is increased in the gut of Akita mice with a similar duration of diabetes [8, 55]. In the current study, we found a significant increase in expression of PV-1 in the small intestine of *db/db* mice with 10 months of diabetes duration (Fig. 4d, f). To confirm these findings, we performed western blots for E-cadherin and PV-1 in small intestine tissue lysates. We observed significantly decreased expression of E-cadherin and significantly increased expression of PV-1 (Fig. 4g–i; ESM Fig. 3). Collectively, our data support the notion that the intestine in type 2 diabetes displays loss of both epithelial and endothelial barrier stability. There appears to be fluctuations in the stability of these barriers throughout diabetes duration, although by late-stage disease both barriers are compromised. Our data suggest that the increased NETosis is associated with generalised barrier disruption but a particular barrier (endothelial vs epithelium) does not appear more vulnerable to the effects of NETosis.

Previously, we have shown that gut barrier permeability and resultant endotoxaemia exacerbate endothelial damage in the retina and participate in the development and progression of diabetic retinopathy in Akita mice [8, 42]. We hypothesised that a similar effect would occur in *db/db* mice. Thus, we performed immunofluorescent staining of VE-cadherin and enumeration of acellular capillaries in retinal flat mounts of *db/db* mice with 10 months of diabetes duration. Of important note, retinal VE-cadherin exhibited gaps in staining, indicating areas of enhanced permeability (denoted by white arrows in Fig. 4j). We found that the number of acellular capillaries were significantly increased in *db/db* mice with 10 months of diabetes duration (Fig. 4j, k). To assess whether these two endpoints may be connected, we performed correlation analysis between intestinal neutrophil abundance and retinal acellular capillary numbers and found that there was a positive correlation in *db/db* mice with 10 months of diabetes duration (Fig. 4l). To further investigate this association, we next pharmacologically inhibited NETosis in *db/db* mice and asked if it would prevent loss of gut barrier integrity, maintain low levels of endotoxaemia and delay onset of diabetic retinopathy in *db/db* mice.

### Global PAD4 inhibition in diabetic mice decreases intestinal indoleamine 2,3-dioxygenase expression, promotes anti-inflammatory profiles and increases abundance of vascular reparative cells in the small intestine and central myeloid pools

**Fig. 5:**
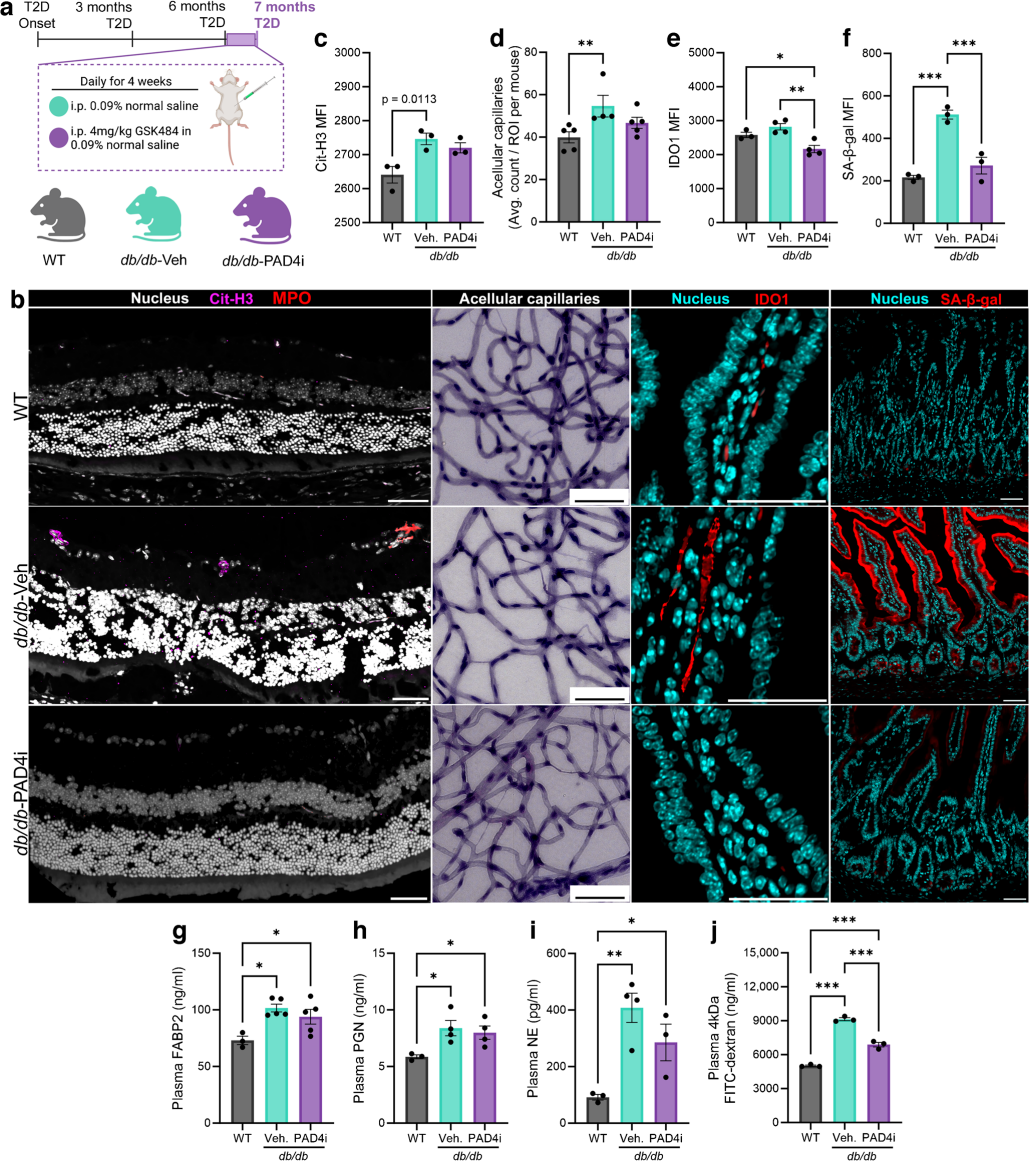
Inhibition of PAD4 reduces retinal NETosis and slows progression of diabetic retinopathy in conjunction with decreasing gut leakage and neutrophilic inflammation. (**a**) To assess the impact of global NETosis inhibition on diabetic retinopathy pathogenesis, gut permeability and systemic inflammation, male *db/db* mice with 6 months of type 2 diabetes duration (7 months age) were randomly divided into two groups (*n*=5 each); age-matched WT mice served as non-diabetic, non-injected controls. Over the course of 4 weeks, starting at 6 months of type 2 diabetes duration, *db/db* mice received daily i.p. injections (100 μl) of either vehicle (*db/db*-Veh, 0.9% normal saline) or the selective, reversible PAD4i GSK484 (*db/db*-PAD4i, 4 mg/kg in 0.9% normal saline). (**b**) Representative images are shown: retinal Cit-H3 and MPO; retinal acellular capillaries, the hallmark histopathological characteristic of murine diabetic retinopathy; and IDO1, a marker of intestinal inflammation. (**c**–**f**) Quantification of retinal Cit-H3 expression (**c**), retinal acellular capillaries (**d**), intestinal IDO1 (**e**) and senescence-associated β-galactosidase (**f**). (**g**–**i**) Quantification of plasma FABP2 (**g**), PGN (**h**) and neutrophil elastase (**i**) as determined by ELISA. (**j**) Quantification of plasma 4 kDa FITC–dextran concentration. Each data point represents one independent observation from one experimental mouse (*n*=3–5 mice per group). *p<0.05, **p<0.01, ***p<0.001. Scale bars, 50 µm. mo, months; NE, neutrophil elastase; SA-β-gal, senescence-associated β-galactosidase; T2D, type 2 diabetes; Veh., vehicle

NETosis is dependent on post-translational modification of histone H3, which requires the activity of peptidyl arginine deiminase 4 (PAD4). PAD4 functions in a calcium-dependent manner to deaminate H3 arginine residues, converting them to the non-classical amino acid citrulline and inducing chromatin decondensation. The activity of PAD4 also induces breakdown of the nuclear envelope, allowing cytoplasmic granule proteins, such as MPO and neutrophil elastase, to bind nuclear DNA and decorate the forming NET. To confirm the specific effect of NETosis on barrier function and endotoxaemia, littermates of *db/db* mice were randomly divided into two cohorts, one of which received i.p. injections of saline vehicle (*db/db*-Veh) and the other received the selective, reversible PAD4i GSK484 (*db/db-*PAD4i) daily for 4 weeks. WT littermates were included as non-diabetic, non-injected control animals. Administration of PAD4i was initiated in *db/db* mice at 6 months of type 2 diabetes (8 months of age) and terminated 4 weeks later, at 7 months of type 2 diabetes (9 months of age) (design detailed in Fig. 5a). We first aimed to confirm previous reports that PAD4 inhibition reduces NETosis within the retina. Therefore, we performed IF staining of Cit-H3 and MPO in retinal cross-sections and found significantly increased Cit-H3 MFI in the retina of *db/db*-Veh mice compared with WT mice (Fig. 5b, c). Cit-H3 expression was increased in *db/db*-PAD4i mice compared with *db/db*-Veh mice, though not to a statistically significant level. We assessed severity of diabetic retinopathy in diabetic mice by enumeration of retinal acellular capillaries. As expected, *db/db*-Veh mice exhibited a significantly increased number of acellular capillaries compared with WT mice (Fig. 5b, d). In *db/db*-PAD4i mice, the number of acellular capillaries exhibited a modest decrease compared with *db/db*-Veh mice but the count remained above that of WT mice, suggesting that 1 month of PAD4i administration may slow the progression of diabetic retinopathy in *db/db* mice (Fig. 5b, d; lower magnification images are shown in ESM Fig. 4a).

Tryptophan, an essential amino acid, is the substrate for indoleamine 2,3-dioxygenase (IDO) 1, with 95% of tryptophan being degraded through the kynurenine pathway by IDO [56, 57]. Previously, we showed that IDO expression in retinal microglia was markedly increased and promoted neuronal degeneration in diabetic retinopathy [58]. Elevated IDO activity has also been implicated in exacerbation of gut permeability and endotoxaemia in models of obesity [59] and the kynurenine pathway has been implicated in T cell dysfunction; however, the impact of IDO and kynurenine on neutrophil function has not been well studied [60]. To determine whether PAD4 inhibition influences IDO expression and metabolism of tryptophan in *db/db* mice, we examined IDO expression by IF and concentrations of tryptophan metabolites within the small intestine by MS of tissue lysates. We found significantly reduced expression of IDO in the small intestine of *db/db-*PAD4i mice compared with both *db/db-*Veh and WT mice (Fig. 5b, e; lower magnification images are available in ESM Fig. 4b). The concentration of Trp was significantly decreased in both *db/db* mouse cohorts compared with WT mice and slightly decreased in *db/db*-PAD4i mice compared with *db/db-*Veh mice (ESM Fig. 4c). A similar pattern was observed in the concentration of kynurenine, though differences were not statistically significant (ESM Fig. 4d). No difference was observed in the concentration of quinolinic acid when comparing mouse cohorts (ESM Fig. 4e). These data support the notion that tryptophan metabolism is altered in the diabetic gut and that knockdown of neutrophilic inflammation by PAD4 inhibition reduced IDO expression.

We next evaluated the impact of PAD4 inhibition on senescence in the intestine. We found SA-β-galactosidase to be significantly increased in *db/db*-Veh mice compared with WT mice and significantly decreased in *db/db*-PAD4i mice compared with *db/db*-Veh mice (Fig. 5b, f). However, at this late stage of disease, PAD4i treatment for 1 month had a negligible effect on plasma FABP2 and PGN in *db/db* mice, though slight decreases in these plasma proteins were observed in *db/db-*PAD4i mice compared with *db/db*-Veh mice (Fig. 5g, h). For assessment of global neutrophilic inflammation, we performed ELISA for plasma neutrophil elastase. We found it to be significantly increased in the plasma of *db/db*-Veh mice with a non-significant improvement in *db/db*-PAD4i mice (Fig. 5i). Additionally, we assessed small intestine permeability through the FITC–dextran assay. In this assay, a 4 kDa FITC-conjugated dextran is orally gavaged to experimental animals and plasma FITC absorbance is assessed 1 h after gavage. This 1 h post-gavage assessment is ideal for determining permeability of the small intestine barrier compared with the colon, which is assessed up to 4 h post-gavage [61]. We found plasma FITC–dextran to be significantly decreased in *db/db*-PAD4i mice compared with *db/db*-Veh mice, though the plasma concentration of FITC–dextran was still significantly higher in *db/db*-PAD4i mice compared with WT mice (Fig. 5j). Collectively, these results support that short-term systemic PAD4i treatment showed benefit by reducing retinal NETosis, slowing progression of diabetic retinopathy, decreasing intestinal inflammation as determined by IDO expression, lowering systemic neutrophil activation and decreasing permeability of the small intestine. We next asked what effect PAD4 inhibition had, specifically on the neutrophil compartment.

### PAD4i promotes resolution of intestinal neutrophilia and retention of pre-neutrophils in the bone marrow while increasing the production and homing of MACs to the gut

**Fig. 6:**
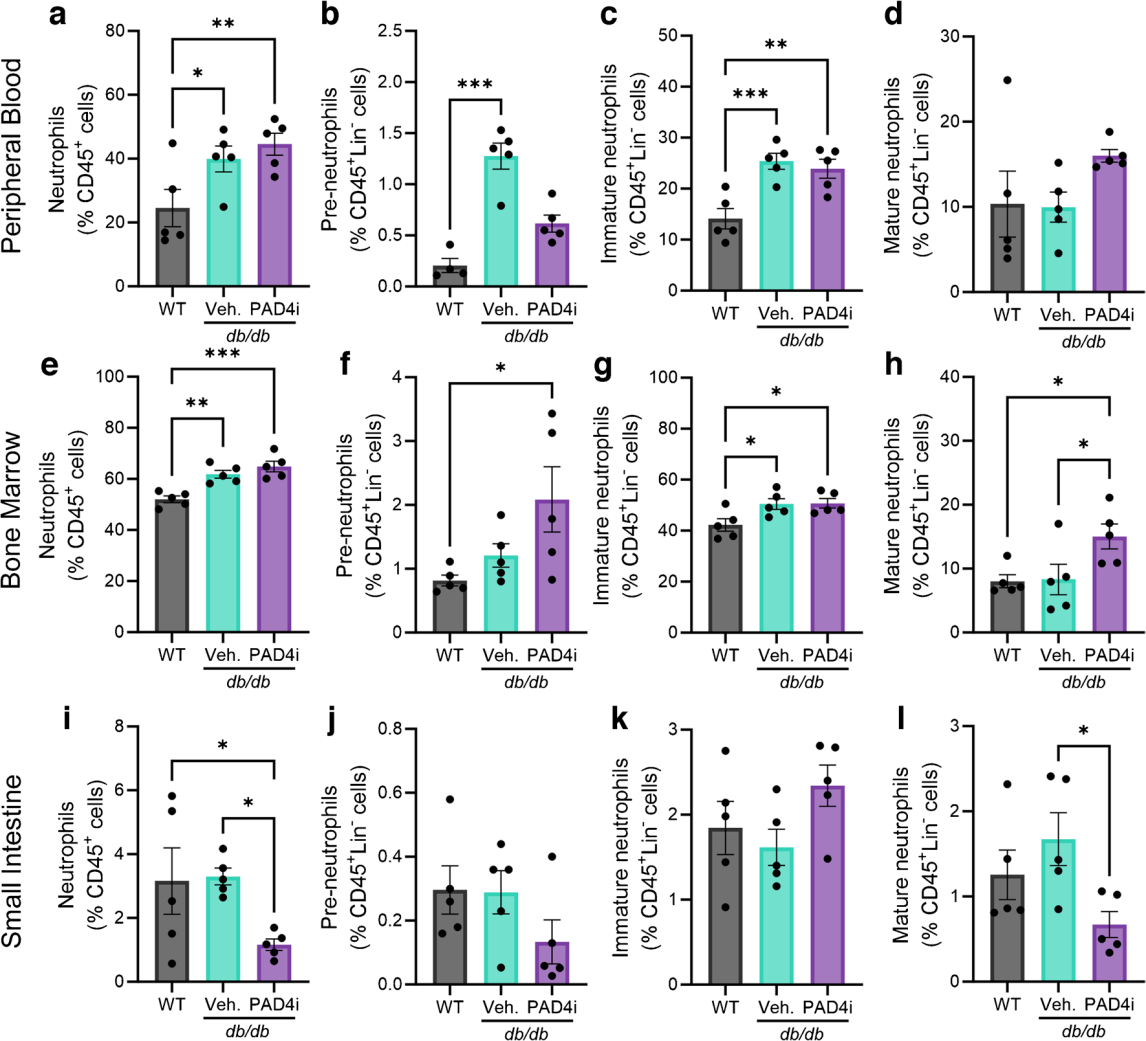
PAD4 inhibition decreased the infiltration of mature neutrophils into the small intestine and retention of pre-neutrophils in the bone marrow. We used flow cytometry to assess the impact of PAD4 inhibition on the abundance of neutrophil subtypes and vascular reparative cells within the peripheral blood, bone marrow and small intestine. (**a**–**d**) Quantification of the abundance of total neutrophils (**a**), pre-neutrophils (**b**), immature neutrophils (**c**) and mature neutrophils (**d**) within the peripheral blood. (**e**–**l**) The quantification was replicated for the bone marrow (**e**–**h**) and the small intestine (**i**–**l**). Each data point represents one independent observation from one experimental mouse (*n*=5 per group). *p<0.05, **p<0.01, ***p<0.001

We next interrogated the effects of PAD4 inhibition on the neutrophil compartment and assessed the distribution between the lineage-committed neutrophil subpopulations and mature neutrophils. Flow cytometry was used, similar to the approach described by Evrard et al [62]. In this manner, CD11b^+^Gr1^+^ neutrophils are classified based on their surface expression of the stem-cell marker cKit (CD117), which identifies those cells that are the most primitive, stem-like but lineage-committed neutrophil populations (pre-neutrophils). Additionally, we examined C-X-C motif receptor 2 (CXCR2) and C-X-C motif receptor (CXCR4) expression, which allows for the separation of cells that are licensed for egress from the bone marrow (CXCR2^+^) vs those which are not licensed and are retained in the bone marrow (CXCR4^+^). Three lineage-committed neutrophil subtypes were identified (Fig. 6): (1) pre-neutrophils (CD11b^+^Gr1^var^Ly6G^+^ cKit^+^CXCR4^+^); (2) immature neutrophils (CD11b^+^Gr1^var^Ly6G^+^cKit^−^CXCR4^var^CXCR2^−^); and (3) mature neutrophils (CD11b^+^Gr1^var^Ly6G^+^ cKit^−^CXCR4^var^CXCR2^+^). Non-mature neutrophils are licensed for bone marrow egress by loss of surface CXCR4 and gain of surface CXCR2 expression and are deemed a hyper-reactive phenotype. This is postulated to be the consequence of incomplete antigen education and decreased antigen tolerance resulting from premature egress. Since the gut contains the largest abundance of neutrophil-activating antigens, we postulated that increased infiltration of pre-neutrophils into the intestine may predispose the gut to neutrophilic damage, especially when compounded with type 2 diabetes-associated inflammation. The abundance of MACs or their recruitment to the gut was considered as an assessment of the degree of vascular damage as the function of these cells is vascular repair.

In the peripheral blood, we observed significantly elevated total neutrophils in both *db/db* mouse cohorts compared with WT mice (Fig. 6a). Pre-neutrophils were increased in the peripheral blood of *db/db*-Veh mice compared with WT mice, with a non-significant decrease in *db/db*-PAD4i mice (Fig. 6b). Immature neutrophils in the peripheral blood of *db/db* mice were unaltered by PAD4i compared with vehicle treatment but were elevated in both *db/db* cohorts compared with WT mice (Fig. 6c). A non-significant increase in mature neutrophils was observed in *db/db*-PAD4i mice (Fig. 6d). In the bone marrow, their site of production, total neutrophils were elevated in *db/db*-PAD4i and *db/db*-Veh mice compared with WT mice (Fig. 6e). The proportion of pre-neutrophils was highest in the bone marrow of *db/db-*PAD4i mice (Fig. 6f). The abundance of immature neutrophils was increased in the bone marrow of both *db/db* cohorts compared with WT mice and the abundance of mature neutrophils was significantly increased in the bone marrow of *db/db*-PAD4i mice compared with both *db/db*-Veh and WT mice (Fig. 6g, h). In the small intestine, the total proportion of neutrophils was found to be reduced in *db/db*-PAD4i mice compared with either *db/db*-Veh or WT mice (Fig. 6i). We found a non-significant decrease in the abundance of pre-neutrophils (Fig. 6j), a non-significant increase in the abundance of immature neutrophils (Fig. 6k) and a significant decrease in the abundance of mature neutrophils within the small intestine of *db/db*-PAD4i mice compared with *db/db*-Veh mice (Fig. 6l). Together, these data support our hypothesis that PAD4 inhibition would promote retention of pre-neutrophils in the bone marrow thus facilitating maturation of these cells before they enter the circulation. In the intestine, PAD4i treatment resulted in fewer mature neutrophils.

In addition to markers that identify neutrophil maturation state, we included within our flow cytometry panel antibodies against proteins associated with neutrophil functional states. These include the primary granule membrane protein CD63 (LAMP-3), the lysosomal membrane protein CD107a (LAMP-1) and L-selectin (CD62L). Increased expression of CD63 and CD107a on neutrophils is indicative of cells that have undergone degranulation, as these markers are shifted to the cell surface during exocytosis of intracellular granules [63–65]. Increased surface expression of CD62L on neutrophils is indicative of increased activation/migration as CD62L is required for firm adhesion of neutrophils to the vascular endothelium. Increased CXCR4 and CD62L on circulating neutrophils is is indicative of aged cells [66].

**Fig. 7:**
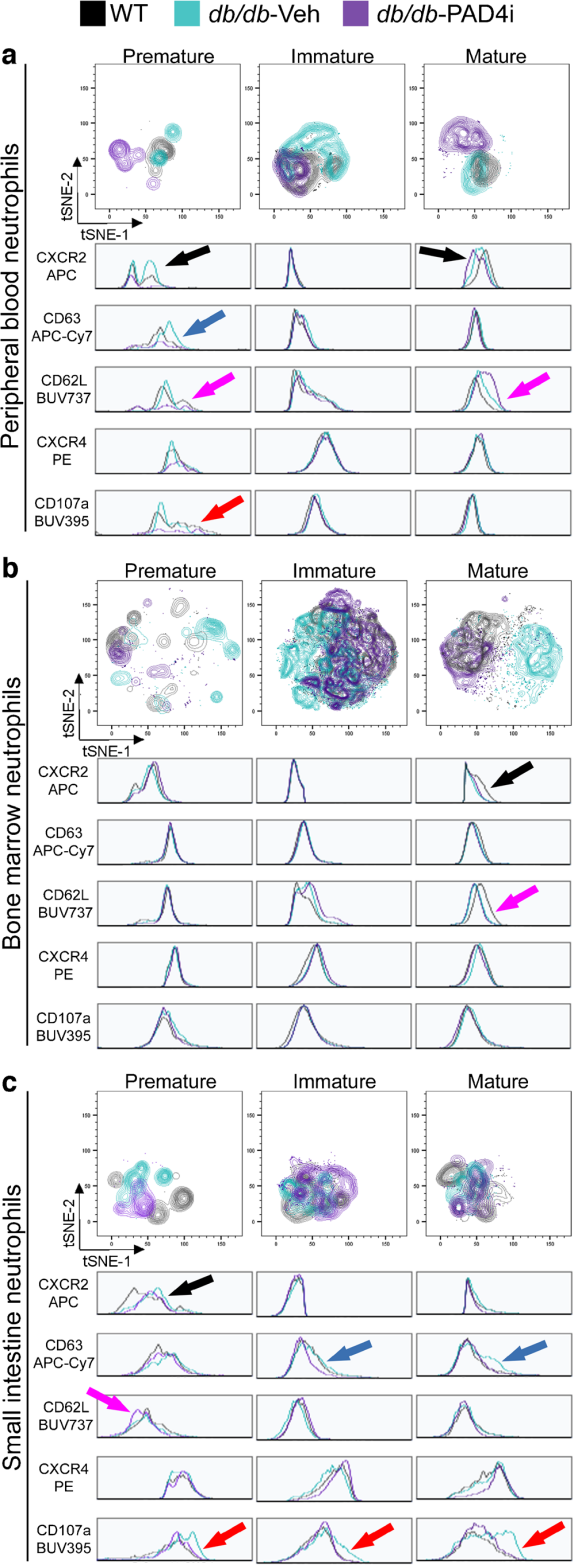
The effect of PAD4 inhibition on functionality of diabetic neutrophils. Flow cytometric analyses displaying community dynamics and function with t-distributed stochastic neighbour embedding (tSNE) plots and histograms. Data are presented for peripheral blood, bone marrow and small intestine neutrophil populations. tSNE plots were created with all markers in the neutrophil gating strategy (CD45, CD11b, Gr-1, cKit, Ly6-G, CXCR2 and CXCR4), markers of activation/degranulation (CD63, CD107a) and a marker of migration (CD62L). Histograms display the geometric MFI of functionality-related surface markers. (**a**) In the peripheral blood, pre-neutrophils exhibited reduced CXCR2 (black arrow), CD63 (blue arrow), CD107a (red arrow) and CD62L (magenta arrow) in *db/db*-PAD4i mice. Mature neutrophils of *db/db*-PAD4i mice exhibited decreased CXCR2 (black arrow) and elevated CD62L (magenta arrow) expression. (**b**) In the bone marrow, mature neutrophils of *db/db*-PAD4i mice exhibited reduced CXCR2 (black arrow) and CD62L (magenta arrow) expression. (**c**) In the small intestine, pre-neutrophils of *db/db*-PAD4i mice exhibited reduced CXCR2 (black arrow), CD62L (magenta arrow) and CD107a (red arrow) expression. Immature and mature neutrophils of *db/db*-PAD4i mice exhibited reduced CD63 (blue arrow) and CD107a (red arrow) expression

In the blood, pre-neutrophils were found to exhibit increased CXCR2 expression in *db/db*-Veh mice compared with *db/db*-PAD4i mice whereas *db/db*-PAD4i mice exhibited decreased expression of CXCR2 in pre-neutrophils and mature neutrophils (Fig. 7a, black arrows). Pre-neutrophils of *db/db*-PAD4i mice exhibited decreased CD63 and CD107a expression compared with *db/db*-Veh mice (Fig. 7a, blue and red arrows, respectively). The expression of CD62L was observed to be decreased in pre-neutrophils and increased in mature neutrophils of *db/db*-PAD4i mice compared with *db/db*-Veh mice (Fig. 7a, magenta arrows). As expected, mature neutrophils within the bone marrow were found to have decreased expression of CXCR2 (Fig. 7b, black arrow) and CD62L (Fig. 7b, magenta arrow) in diabetic mice, irrespective of PAD4i treatment status, compared with WT mice. In the intestine, we found that pre-neutrophils of diabetic mice exhibited increased expression of CXCR2 compared with WT mice, irrespective of PAD4i treatment (Fig. 7c, black arrow). Immature neutrophils of *db/db*-PAD4i mice were observed to express less CD63 than *db/db*-Veh and WT mice (Fig. 7c, blue arrow). Conversely, mature neutrophils of *db/db*-Veh mice exhibited higher expression of CD63 than *db/db*-PAD4i and WT mice (Fig. 7c, blue arrow). *db/db*-Veh mice exhibited increased expression of CD107a in all three neutrophil populations within the intestine compared with WT and *db/db*-PAD4i mice (Fig. 7c, red arrows). These data support the notion that PAD4 inhibition reduced the degranulative potential of the entire intestinal neutrophil compartment and suggest that PAD4 inhibition reduced the migratory capacity of pre-neutrophils in the circulation.

Collectively, these data support the notion that PAD4i administration to diabetic mice results in decreased activation and degranulation of neutrophils in the intestine. Because neutrophils do not work alone in guarding the body from gut-derived pathogens and attract other immune populations (monocytes and macrophages) and vascular reparative cells (circulating angiogenic cells [CACs] and MACs), we next examined these populations.

### PAD4 inhibition promotes expansion of vascular reparative cells and their migration to the gut

To assess the abundance of the other myeloid cell populations, we analysed the proportions of macrophages, monocytes and angiogenic cells in the blood, bone marrow and gut from the three mouse cohorts. In the peripheral blood, total monocytes (CD11b^+^Ly6G^−^Ly6C^+^; ESM Fig. 5a), classical (CCR2^+^; ESM Fig. 5b) and non-classical (CCR2^−^; ESM Fig. 5c) monocytes were increased in abundance in *db/db*-PAD4i mice compared with *db/db*-Veh mice. PAD4i treatment of the *db/db* mice resulted in changes in two populations of haematopoietic cells that foster vascular repair: MACs; and CACs. Levels of MACs (CD11b^+^Ly6G^−^Ly6C^−^F4/80^−^Flk1^+^CD31^+^; ESM Fig. 5d) and CACs (CD11b^−^Flk1^+^CD31^+^; ESM Fig. 5e) were increased in the blood of *db/db*-PAD4i mice compared with *db/db*-Veh mice, with levels resembling those in WT mice. However, the change in the levels of CACs did not reach statistical significance. In the bone marrow, we detected an increase abundance of total monocytes (ESM Fig. 5f) and classical monocytes (ESM Fig. 5g) in *db/db*-PAD4i mice compared with WT controls. In all cases except for non-classical monocytes (ESM Fig. 5h), the proportion of these populations were increased (non-significantly) compared with *db/db*-Veh littermates. In bone marrow, the abundance of MACs and CACs was found to be significantly increased in *db/db*-PAD4i mice compared with *db/db*-Veh mice (ESM Fig. 5i, j). The abundance of intestinal macrophages (CD11b^+^Ly6G^−^Ly6C^var−^F4/80^+^) was decreased in *db/db*-PAD4i mice due to a decrease (non-significant) in inflammatory M1 (CD206^−^) macrophages and a significant decrease in anti-inflammatory M2 (CD206^+^) macrophages (ESM Fig. 5k–m). The increased infiltration of MACs in *db/db*-PAD4i mice was significant compared with *db/db*-Veh mice and non-significant compared with WT mice. The increased infiltration of CACs in *db/db*-PAD4i mice was not significant (ESM Fig. 5o) compared with *db/db*-Veh and WT mice. These data lend support for global benefits of PAD4 inhibition on vascular repair processes, specifically by increasing the abundance of cell populations that promote vascular repair in a paracrine manner [8].

Originating from the most primitive long-term repopulating haematopoietic stem cells (LT-HSCs, Lin^−^ Sca1^+^cKit^+^[LSK] Flt3^−^CD34^−^), neutrophils are the product of a multi-stage differentiation programme that includes at least five intermediate populations prior to lineage commitment. We characterised the abundance of these distinct populations in the bone marrow and found there to be no change in the abundance of LT-HSCs (ESM Fig. 6a) but there was a significant increase in the proportion of short-term repopulating haematopoietic stem cells (LSK Flt3^−^CD34^+^) in *db/db*-PAD4i mice compared with both *db/db*-Veh and WT mice (ESM Fig. 6b). Significant modulations in other intermediates were not observed when comparing *db/db*-PAD4i and *db/db*-Veh mice, though we observed a non-significant decrease in multipotent progenitors (LSK Flt3^+^CD34^+^; ESM Fig. 6c) and in megakaryocyte–erythroid progenitors (MEPs, Lin^−^Sca1^−^cKit^+^[LS^−^K] FcγRII/III^−^CD34^−^; ESM Fig. 6f) and non-significant increases in common myeloid progenitors (LS^−^K FcγRII/III^−^CD34^+^; ESM Fig. 6d) and granulocyte–monocyte progenitors (LS^−^K FcγRII/III^+^CD34^+^; ESM Fig. 6e). Together, these data translated to elevated abundance of myeloid effector populations and vascular reparative cells within the peripheral blood and small intestine, suggesting that, indeed, inhibition of NETosis may promote vasculature repair either indirectly through modulation of inflammatory homeostasis or directly by increasing key reparative populations.

## Discussion

The major findings of this study include observations of neutrophilia and increased NLR in individuals with type 2 diabetes with advanced NPDR and DME compared with individuals with type 2 diabetes without diabetic retinopathy and age- and sex-matched healthy individuals. Intriguingly, the presence of DME and NPDR was associated with the highest neutrophil counts and the greatest NLR, suggesting that neutrophilia may be particularly critical to the formation of DME. Elevated levels of gut permeability and endotoxaemia markers were observed in individuals with type 2 diabetes and were even higher in those with advanced diabetic retinopathy and DME. These data lends support to the notion that peripheral neutrophilic inflammation is associated with a hyper-permeable gut and exacerbated endotoxaemia and may play a role in the development of DME. In the *db/db* mouse model of type 2 diabetes but not the Akita mouse model of type 1 diabetes we found systemic and intestinal neutrophilia.

These data agree with our previous findings that demonstrated that gut inflammation is not a characteristic of type 1 diabetes in Akita mice, though a reduction in intestinal MACs was observed in the models of both type 1 and type 2 diabetes we studied. In fact, we were able to correct gut barrier dysfunction and reduce endotoxaemia in Akita mice through i.p. administration of MACs isolated from WT mice. In contrast, we observed inflammation in type 2 diabetes as demonstrated by multi-site neutrophilia herein. Within the gut of *db/db* mice, neutrophils were found to have increased capacity for NETosis and degranulation, confirming in vitro and ex vivo phenotypes of diabetic neutrophils. This study, to our knowledge, represents the first in vivo study of intestinal NETosis in the context of diabetes.

Increased neutrophils are associated with acute and intermittent hyperglycaemia. Neutrophils release alarmins S100A8 and S100A9, which bind to TLR-4 and prime the nod-like receptor family pyrin domain-containing 3 inflammasome in naive neutrophils and promote IL-1β secretion. The released IL-1β interacts with its receptor (IL-1 receptor type 1) on haematopoietic stem cells and progenitor cells in the bone marrow and stimulates granulopoiesis in a cell-autonomous manner [67]. While this behaviour was shown for myocardial induced granulopoiesis, it can be seen with other stressors such as type 2 diabetes. In individuals with acute coronary syndrome, higher neutrophil count on hospital admission and after revascularisation correlates positively with major adverse CVD outcomes [67]. Thus, neutrophilia and neutrophil-derived alarmins (S100A8 and S100A9) dictate the nature of the ensuing inflammatory response after injury. Therapeutic strategies to reduce neutrophilia and NETosis are aimed at disruption of S100A8/S100A9 signalling or their downstream mediators in neutrophils, thus suppressing excessive granulopoiesis as seen in type 2 diabetes.

NETs in the setting of diabetes can also function as a stimulus for NLR family pyrin domain-containing 3 (NLRP3) inflammasome activation in macrophages to promote IL-1β-dependent exacerbation of inflammation. These findings provide insight into how NETs communicate with other cells in the vicinity (e.g. macrophages) to exacerbate the inflammatory response [68].

ROS production is an important driver of neutrophilia [69]. Type 2 diabetes mellitus is accompanied by halogenated stress as neutrophilic MPO generates highly reactive hypochlorous acid (HOCl), which can modify serum albumin (Cl-HSA). Cl-HSA induces neutrophil priming and NETosis. Accelerated NET degradation and neutrophil priming can contribute to the development of complications in type 2 diabetes [70].

The significance of neutrophilia to diabetic retinopathy is that these cells are recruited to the microvascular beds of the retina where they can cause damage to the endothelium. Neutrophils respond to CXCL1 through activation of CXCR2, and this cytokine is elevated in the retinas of individuals with diabetic retinopathy [71]. Notably, the intravitreal administration of CXCL1 leads to increased retinal vascular permeability accompanied by neutrophil recruitment [71]. Once these cells appear in the retina, they can contribute to development of diabetic retinopathy [22, 72]. Studies have shown increased NET levels in the serum and retinas of diabetic individuals, possibly due to NADPH oxidase activation [72]. NETosis, which plays a role in removing senescent vasculature, may contribute to vascular remodelling and angiogenesis in diabetic retinopathy [22]. However, the *db/db* mouse model does not display neovascularisation and our use of NET inhibitor was short term, explaining the non-significant change in the number of acellular capillaries in the *db/db*-PAD4i mice compared with *db/db*-Veh and WT mice. If we had continued the PAD-4i treatment for longer, we may have observed a significant reduction in acellular capillary formation. Additionally, if we had started the PAD4i at the onset of diabetes we may have been able to prevent the development of diabetic retinopathy. Early NETosis inhibition has shown promising results. Previously, genetic knockout of neutrophil elastase in diabetic mice protected retinal vessels from leakage and degeneration in the early stages of diabetic retinopathy [17].

Previous work from our group has implicated a hyper-permeable gut barrier and subsequent endotoxaemia in the progression and severity of diabetic retinopathy in humans and mouse models of type1 diabetes [42]. In the current studies, we aimed to characterise the intestinal neutrophil compartment in a mouse model of type 2 diabetes and determine whether the heightened neutrophil activity observed promoted gut barrier disruption, endotoxaemia and diabetic retinopathy progression. Neutrophils are major players in barrier immunity, especially in the gut where bacterial abundance is at its highest in the body. Though the bactericidal programmes employed by neutrophils to clear infectious infiltrates are tightly controlled in health, gut dysbiosis and chronic, rather than acute, inflammation (such as is present in type 2 diabetes) induces a baseline ‘primed’ phenotype of the neutrophil compartment. This causes neutrophil function to be exaggerated relative to that of non-diabetic neutrophils. Diabetic neutrophils exhibit increased propensity for NETosis, degranulation, extracellular ROS production and inflammatory cytokine secretion but have a decreased ability for chemotaxis, phagocytosis and apoptosis. In health, pre-neutrophils are restricted to the bone marrow and mature neutrophils predominate in the circulation and in tissue-infiltrating pools. In this type 2 diabetes study, we showed that pre-neutrophils are more likely to be found in the circulation and peripheral tissues. Thus, heightened pre-neutrophil abundance in a target tissue is indicative of an elevated inflammatory milieu, which results in a form of emergency granulopoiesis to force pre-neutrophils into action. Due to the nature of their premature egress from the bone marrow, pre-neutrophils experience less ‘neutrotime’ (the time undergone between lineage commitment and egress into the circulation) and, thus, they have decreased antigen education and tolerogenic capacity, which promotes their hyper-reactive phenotypes. Further, pre-neutrophils are at a point in maturation in which they lack secondary and tertiary granules, those which contain many of the pro-resolving proteins, so any damage inflicted during their response has decreased propensity for physiological healing.

NETosis carries a great risk of concomitant tissue damage as it typically results in lytic cell death of the neutrophil and release of cytoplasmic stores of cytotoxic granule proteins and ROS. Given mounting evidence that suggests neutrophils and NETosis contribute to microvascular complications of diabetes in the intestine and retina, we asked whether pharmacological inhibition of NETosis for 4 weeks (between 6 and 7 months of diabetes duration) in *db/db* mice would promote a pro-resolving phenotype in diabetic neutrophils and decrease the abundance of pre-neutrophils in the circulation and intestine. We convincingly show that short-term inhibition of NETosis via i.p. administration of the selective, reversible PAD4 antagonist GSK484 results in a decreased abundance of pre-neutrophils and mature neutrophils in the small intestine while promoting an anti-inflammatory myeloid phenotype, recruitment of protective MACs to the gut and retention of stem-like pre-neutrophils in the bone marrow. Neutrophils of *db/db-*PAD4i mice exhibited decreased expression of activation and degranulation markers and presented a ‘mature’ phenotype compared with those treated with vehicle. Gut permeability was reduced in *db/db* mice receiving PAD4i. In the retina, while we did not observe a significant reduction in the number of acellular capillaries (a marker of vasodegeneration) in *db/db*-PAD4i mice compared with *db/db*-Veh mice, we did observe a decrease in IDO staining supporting reduced inflammation.

This study had limitations. We did not perform in vitro functional studies using neutrophils. The isolation of neutrophils for in vitro studies is extremely problematic. Neutrophils have a very short t½ in the blood (only 6–8 h) and after purification from the blood the cells quickly go to apoptosis [73, 74]. Neutrophils do not proliferate, making it impossible to expand them in vitro, and cryopreservation of these cells has so far not proven successful [75]. Thus, our focus was to determine their behaviour in vivo. We enumerated neutrophils and phenotyped their activation state in tissues of interest using flow cytometry and determined their location and activation state using immunohistochemistry. Some of our PAD4i data in the retina approached but did not achieve significance. There are no studies using PAD4i in rodents for longer than 1 month that we could identify. Thus, we reasoned that the chronic use of PAD4i could result in immune compromise and unwanted pathology that would confound the interpretation of the beneficial effects we had hoped to see. Thus, we elected to terminate our PAD4i experiments at the maximum timepoint found in previous studies (i.e. 1 month of daily injections). However, a longer time course of PAD4i treatment could have resulted in significant changes. Finally, while we show that a disrupted gut barrier leads to progressive and consistent endotoxaemia (10 months), beneficial compensatory mechanisms may come into play at earlier time points of diabetes. In the current study, we did not explore the impact of the microbiome or diet on NETosis, nor did we determine whether use of PAD4i could have altered the microbiome. Our study mice were all male and thus we cannot conclude that our results would apply to female mice.

This study and others have demonstrated beneficial effects of NETosis inhibition in diabetic rodents; a similar pharmacological approach is not likely to be accepted in human studies since neutrophils are so important for immune responses that diminishing their function carries risk for infection. Therefore, more targeted approaches are necessary if modulation of neutrophil activity is the desired therapeutic approach. Approaches that target the microbiome to reduce the extent of meta inflammation in diabetes may provide a more reasonable path to indirectly modulating neutrophil activity for improving vascular outcomes.

In summary, our findings confirm neutrophilia in type 2 diabetes and stratifies diabetic individuals by the presence/absence of diabetic retinopathy, supporting the notion that neutrophilia with increased abundance of pre-neutrophils and increased NLR are associated with gut barrier dysfunction and the presence of NPDR and DME. Thus, these findings suggest that diabetic neutrophilia and neutrophil hyperactivation are associated with worsened microvascular outcomes that are due to loss of gut barrier integrity and endotoxaemia.

## Supplementary Information

Below is the link to the electronic supplementary material.

ESM (PDF 2365 KB)

## Data Availability

All data supporting the findings of this study are available within the paper and its ESM.

## Abbreviations

CAC: Circulating angiogenic cell
Cit-H3: Citrullinated histone 3
CXCR2: C-X-C motif receptor 2
CXCR4: C-X-C motif receptor 4
DME: Diabetic macular oedema
E-cadherin: Epithelial cadherin
FABP2: Fatty acid binding protein 2
FFPE: Formalin-fixed paraffin-embedded
GMA: Gut-derived microbial antigen
IDO: Indoleamine 2,3-dioxygenase
IF: Immunofluorescence
LBP: Lipopolysaccharide-binding protein
LT-HSC: Long-term repopulating haematopoietic stem cell
MAC: Myeloid angiogenic cell
MFI: Mean fluorescence intensity
MPO: Myeloperoxidase
NET: Neutrophil extracellular trap
NLR: Neutrophil/lymphocyte ratio
NPDR: Non-proliferative diabetic retinopathy
PAD4: Peptidyl arginine deiminase 4
PAD4i: Peptidyl arginine deiminase 4 inhibitor
PDR: Proliferative diabetic retinopathy
PGN: Peptidoglycan
PFA: Paraformaldehyde
PV-1: Plasmalemma vesicle-associated protein 1
ROS: Reactive oxygen species
TLR: Toll-like receptor
VE-cadherin: Vascular endothelial cadherin
WT: Wild-type
ZO-1: Protein zonula occludens 1
