# Supplementary material for: Intestinal neutrophil extracellular traps promote gut barrier damage exacerbating endotoxaemia, systemic inflammation and progression of diabetic retinopathy in type 2 diabetes
Source: Diabetologia. 2025 Jan 28;68(4):866–89. doi: 10.1007/s00125-024-06349-4 (PMC11950064; doi:10.1007/s00125-024-06349-4)
Supplement: Supplementary file 1 — ESM (PDF 2365 KB) [file 125_2024_6349_MOESM1_ESM.pdf]

Electronic Supplementary Material

Extended Methods:

Human participants

Blood was collected at the UAB Callahan Eye Hospital and Clinics according to Institutional Review Board (IRB) guidelines and under the approval of IRB protocols 300000173, 300000068 and 300000188. Whole blood was collected from individuals with type 2 diabetes with varying severities of diabetic retinopathy and DME. Diabetic retinopathy severity was assessed by fellowship trained retinal specialists via fundus examination and graded according to the diabetic retinopathy international classification system [44]. Presence or absence of DME was determined by spectrum domain optical coherence tomography. Eligibility criteria included that study subjects did not have an acute or chronic infection at the time of venipuncture, no ongoing malignancy, not pregnant, no history of organ transplant, no graft, no uremic symptoms and no anemic. The study subjects also did not have any of the following eye conditions: AMD, glaucoma, uveitis, known hereditary degenerations, or other significant ocular complications except for diabetic retinopathy. Blood was collected from healthy control individuals without eye disease. Individuals were matched ± 5 years of age. All blood was placed into EDTA-coated plasma tubes and non-EDTA-coated serum tubes. All blood collections occurred during the morning hours to reduce possible circadian-related variations in immune and metabolic outcomes. Plasma and serum samples were stored at −80°C until processed. A total of 59 healthy control individuals, 27 individuals with type 2 diabetes without eye disease, and 31 individuals with type 2 diabetes with diabetic retinopathy ± DME were assessed for abundance of peripheral immune cells, markers of metabolic dysfunction and markers of gut permeability/endotoxemia. Demographic characteristics, relevant biological variables, clinical interventions and individual stratification by cohort are included in Table 1.

Human peripheral blood flow cytometry

Antibodies used in flow cytometry studies can be found in ESM Table 1.
Whole blood (200µL) from EDTA-coated plasma tubes were lysed in 1mL of ACK lysing buffer (ThermoFisher Scientific, cat.no. A1049201) at room temperature (RT) for 5min after which ACK was neutralized by addition of 1mL FACS buffer (5% fetal bovine serum (FBS) in 1X PBS containing 2.5mM EDTA). Samples were centrifuged at 300g for 5min at 4°C after which supernatant was discarded. If RBCs were incompletely lysed, protocol was repeated. After RBC lysis, cells were washed once in 1mL FACS buffer then centrifuged at 300g for 5min at 4°C. The supernatant was discarded, and the cells resuspended in 200µL FACS buffer and stained for 45min at 4°C in the dark with required flow cytometry antibodies. After staining, samples were washed once in 1mL FACS buffer, centrifuged at 300g for 5min at 4°C, the supernatant discarded, and cells resuspended in 200µL FACS buffer before acquisition. Fluorescence compensations were performed with UltraComp eBeads™ Compensation Beads (Invitrogen, 01-2222-42). Compensation beads were stained in individual tubes for each antibody per panel, staining was performed in the dark for 1 hour at 4°C. Beads were washed once with FACS buffer and acquired. Acquisition and compensations were performed in collaboration with the UAB Comprehensive Flow Cytometry Core. Samples were acquired on a BD Biosciences FACS Celesta cell analyzer and analyzed with FlowJo v10 software.

Housing, metabolic evaluation, and genotyping of experimental mice

Pups generated in the UAB animal facility using an in-house breeding scheme and housed under standard conditions by maintaining a 12:12 hr. light:dark cycle, room temperature of 24±2°C, and 50±10% humidity. Standard chow and water were provided *ad libitum for the duration of all experiments.* Only healthy-appearing mice were used for this study; any mouse

that exhibited evidence of illness, such as excessive wasting or lethargy, was excluded. Cages were not stacked to prevent fecal contamination. Male *Akita* and *db/db* mice exhibit worsened diabetes compared to female littermates; for this reason and due to resource limitations, only male mice were used in these studies. *Akita* mice develop diabetes at ~4-5 weeks of age.[1] Metabolic characteristics and genotyping of *Akita* and *db/db* mice have been previously published.[2] For each experiment, mice from each cohort had a metabolic evaluation (random blood glucose and glycated hemoglobin (HbA1c)) and were confirmed to be diabetic, if applicable, prior to cohort randomization.

Identification of gene status in heterozygous *Akita* and homozygous *db/db* mice was confirmed by genotyping using RT-PCR followed by agarose gel electrophoresis. PCR conditions: *Akita* (Stage I: 94°C for min; Stage II: 94°C for 20sec, 64°C for 30sec, 72°C for 35sec (12 cycles); Stage III: 94°C for 20sec, 28°C for 30sec, 72°C for 35sec (28 cycles); Stage IV: 72°C for 2min) and *db/db* (Stage I: 94°C for 1.5min; Stage II: 94°C for 30sec, 52°C for 45sec, 72°C for 45sec (35cycles); Stage III: 72°C for 2min). The PCR product of *Akita* RT-PCR underwent restriction enzyme digestion for 60min at 37°C (10µL PCR product + 0.5µL Fnu4HI enzyme (BioLabs, cat. no. R0178L, 10000U/mL) + 2.5µL CutSmart Buffer (BioLabs, cat. no. B7204S, 10X) + 7.5µL molecular-grade water). The PCR product of *db/db* RT-PCR underwent restriction enzyme digestion for 60min at 37°C (10µL PCR product + 0.5µL RsaI enzyme (ThermoFisher Scientific, cat. no. ER1121, 1000U/mL) + 2.5µL AB PCR Buffer II (supplied with RsaI enzyme, 10X) + 7.5µL molecular-grade water). Products of restriction enzyme digestion for both *Akita* and *db/db* genotyping were visualized on a 3% agarose gel prepared in 1X Tris-acetate EDTA buffer containing ethidium bromide. Primers utilized for each strain are detailed in ESM Table 2.

<u>Mouse blood and tissue isolation</u>

Mice were anaesthetized with isoflurane prior to cervical dislocation. Whole blood was obtained by cardiac puncture and collected into heparinized tubes; plasma was collected by centrifugation at 2000rpm for 10min at RT post-removal of 100µL aliquots per required flow panel. Plasma was stored at -80°C until further analyzed. From each mouse, femurs and tibias from both hind legs were isolated: one tibia and one femur were used in flow cytometric studies, one tibia flash-frozen for RNA and protein studies, and one femur used for immunofluorescence studies. For assessment of ocular pathology, two eyes from each mouse were enucleated: one eye fixed in 4% paraformaldehyde (PFA) for 24h at 4°C and utilized for staining of acellular capillaries, one eye was fixed in 4% PFA for 30min on ice and utilized for immunofluorescence studies of frozen tissue sections. From each mouse, approximately 4cm of small intestine were resected starting at the most distal portion of the ileum, directly above the cecum. Each 4cm section was flushed with 10mL chilled 1X PBS to remove the luminal contents. The tissue was divided into three pieces: one of which was flash-frozen for protein and RNA studies, the second piece utilized for immunofluorescence studies, and third piece utilized in flow cytometric studies.

<u>Murine flow cytometry studies:</u>

Antibodies used in flow cytometry studies can be found in ESM Table 1 and gating strategies can be found in ESM Figure 5.

Peripheral Blood: 200µL of whole blood was lysed in 1mL of ACK lysing buffer (ThermoFisher Scientific, cat.no. A1049201) at room temperature (RT) for 5min after which ACK was neutralized by addition of 1mL FACS buffer (5% bovine serum albumin (BSA) in 1X PBS containing 2.5mM EDTA. Samples were centrifuged at 300g for 5min at 4°C after which supernatant was discarded. If RBCs were incompletely lysed, protocol was repeated. After RBC lysis, cells were stained for 45min at 4°C in the dark with required flow cytometry antibodies then washed once in FACS buffer before acquisition.

Bone Marrow: The ends of each bone were cut off and cells placed in a 0.5mL Eppendorf tube which was punctured at the bottom with a 21G needle. Each 0.5mL Eppendorf tube containing cut bones was placed inside of a 1.5mL Eppendorf tube containing 200μL FACS buffer and centrifuged at 6000rpm for 1min at 4°C. This technique allows for collection of bone marrow cells without mechanical disruption or activation. The supernatant was collected and stored at -80°C for analysis of protein within the bone marrow interstitial spaces. Cells were resuspended in 1mL FACS buffer and aliquoted in volumes of 100μL per required flow panel, Cells were stained for 45min at 4°C in the dark with required flow cytometry antibodies then washed once in FACS buffer before acquisition.

Small intestine: After flushing, ileum sections were split longitudinally to expose the lumen and placed in a clean 50mL conical tube containing 10mL H2 wash buffer (Ca^{2+} and Mg^{2+} free HBSS containing 2% FBS and 1% Penicillin-Streptomycin (Pen-Strep)) and stored on ice until further processed. Once all samples were collected, each tube was vigorously vortexed for 10sec to remove the remaining luminal contents. Supernatant was discarded and tissue collected into a clean 50mL conical tube containing 20mL of pre-warmed (37°C) Epithelial-Stripping Buffer (H2 wash buffer + 5mM EDTA + 1mM dithiothreitol (DTT) + 1% Pen-Strep). The tissue was incubated in epithelial stripping buffer for 10min at 37°C with gentle rocking. Tubes were then vigorously vortexed for 10sec before being passed through a 40μm nylon filter into a clean 50mL tube. Supernatant was kept on ice and tissue washed in 25mL of pre-warmed H2 wash buffer and vigorously vortexed for 10sec. Supernatant was collected into the 50mL tube containing supernatant from epithelial stripping and tissue was minced with scissors before being placed in digestion buffer (Complete RMPI [RPMI supplemented with 2mM L-glutamine, 1% Pen-Strep, 50mM beta-Mercaptoethanol, 1X non-essential amino acids, 10mM HEPES, 1mM sodium pyruvate, and 10% FBS] + 1mg/mL Collagenase IV + 0.1 U/mL Dispase II + 200 ug/mL DNase I). The tissue was incubated in 10mL digestion buffer for 45min at 37°C with gentle rocking. After digestion, supernatant was collected into tube containing supernatant from previous steps and tissue washed in 10mL of Complete RPMI with vigorous vortexing for 10sec. Supernatant was collected with that of previous steps and remaining tissue discarded. The 50mL tube containing supernatants from previous steps was brought to a final volume of 45mL before being centrifuged at 300g for 5min at 4°C. The supernatant remaining after centrifugation was collected into a new 50mL conical tube and HALT protease inhibitor cocktail (ThermoFisher Scientific, cat.no. 78429) added at a dilution of 1μL per 10mL supernatant. Cells were resuspended in 5mL FACS buffer and centrifuged at 300g for 5min at 4°C to wash off remnant media. The supernatant of this wash was discarded, and cells resuspended in 1mL FACS buffer and aliquoted in volumes of 100μL per required flow panel. Remaining cells were frozen for RNA and protein studies and stored at -80°C until further analyzed. Cells were stained for 45min in the dark at 4°C with required flow cytometry antibodies then washed once in FACS buffer before acquisition.

Fluorescence compensations were performed with UltraComp eBeads™ Compensation Beads (Invitrogen, 01-2222-42). Compensation beads were stained in individual tubes for each antibody per panel, staining was performed in the dark for 1 hour at 4°C. Beads were washed once with FACS buffer and acquired. Acquisition and compensations were performed in collaboration with the UAB Comprehensive Flow Cytometry Core. Samples for all flow cytometry experiments were acquired on either a BD Biosciences FACS Celesta or FACSymphony A5 cell analyzer and analyzed with FlowJo v10 software.

<u>Immunofluorescence staining of murine tissues</u>

Antibodies used in immunofluorescence studies are detailed in ESM Table 3.

FFPF sections: Immunofluorescence studies of FFPE ileum sections were conducted as previously described by our group[2, 3]. Briefly, ileum tissue was fixed in 4% PFA for 2h at 4°C then dehydrated in 70% ethanol before embedding. The tissue was embedded and sectioned by

the Comparative Pathology Lab core facility at UAB. Sections (12μm thick) were deparaffinized with three xylene washes (5min, 5min, 10min) and three washes of 100% ethanol (5min, 5min, 10min). Slides were then rehydrated in 95% ethanol and dH_2O for 5min each. Slides underwent antigen retrieval/unmasking for 45min in 2mm EDTA in PBS at 95°C (pH 8.0) for junctional proteins or sodium citrate buffer in PBS at 70°C (10mM sodium citrate + 0.05% Tween-20, pH 6.0) for neutrophil and NET proteins. Following antigen retrieval, slides were allowed to cool to RT in retrieval buffer before being washed three times in PBS then permeabilized with 0.5% Triton-X100 in PBS for 10 min at RT. Endogenous peroxidase autofluorescence was quenched with 0.3% H_2O_2 in ice-cold methanol for 30min after which slides were again washed in PBS. Slides were blocked in PBS containing 5% horse serum + 2.5% donkey serum + 2.5% goat serum for 1hr at RT. Slides were incubated overnight with desired antibodies at a 1:200μL dilution in blocking buffer at 4°C. Following washing, slides were incubated at RT for 2h in PBS containing secondary antibodies (1:500μL dilution). In all experiments, an isotype control slide which was not incubated in primary antibody (only blocking buffer) was included. After secondary antibody incubation, slides were washed twice in PBS. On the third wash, DAPI (Invitrogen, cat.no. D1306, Waltham, MA) or ToPro3 (Invitrogen, cat.no. T3605, Waltham, MA) was included at a dilution of 1:500μL and incubated at RT for 10min to stain nuclei then mounted in ProLong^{TM} Gold Antifade Mountant (Invitrogen, cat.no. P36930, Waltham, MA) and stored at 4°C in the dark until imaging.

Cryosections: Immunofluorescence of retinal [4, 5] and small intestine cross-sections was performed as previously described. Briefly, eyes and ileum were fixed in 4% PFA for 30min and 2hr, respectively, on ice followed by incubation in 15% and 30% sucrose in 1X PBS, each for 24h, at 4°C. Dehydrated tissues were embedded in optical cutting temperature (OCT) medium, immediately frozen on dry ice, and stored at -80°C until sectioned. Sections were produced by the Comparative Pathology Lab core facility at UAB. Sections (12μm thick) were thawed at 37°C for 20min, rehydrated in PBS, and permeabilized with 0.3% Triton-X100 (retina) or 0.5% Triton-X100 (ileum) in PBS for 10 min at RT. After permeabilization, sections were washed in PBS three times for 5min each after which they were incubated in 0.3% H_2O_2 in ice-cold methanol for 30min to reduce endogenous autofluorescence. They were washed three times in PBS for 5min each then blocked in PBS containing 5% horse serum + 2.5% donkey serum + 2.5% goat serum for 1hr at RT. Slides were incubated overnight with desired antibodies at a 1:200μL dilution in blocking buffer at 4°C. Following washing, slides were incubated at RT for 2h in PBS containing secondary antibodies (1:500μL dilution). In all experiments, an isotype control slide which was not incubated in primary antibody (only blocking buffer) was included. After secondary antibody incubation, slides were washed twice in PBS. On the third wash, DAPI (Invitrogen, cat.no. D1306, Waltham, MA) or ToPro3 (Invitrogen, cat.no. T3605, Waltham, MA) was included at a dilution of 1:500μL and incubated at RT for 10min to stain nuclei then mounted in ProLong^{TM} Gold Antifade Mountant (Invitrogen, cat.no. P36930, Waltham, MA) and stored at 4°C in the dark until imaging.

Senescence staining: In frozen small intestine cross-sections, we performed staining for senescence-associated beta-galactosidase using the CellEvent Senescence Green Detection Kit (Invitrogen, C10851) according to the manufacturer's instructions.

All images in this manuscript were acquired with a Nikon Eclipse Ti2-E inverted microscope with Nikon AX-R confocal module equipped with 405nm, 488nm, 561nm, and 640nm laser lines. All images were analyzed with Nikon NIS-Elements Advanced Research software and graphed using GraphPad Prism v9.1 software.

<u>ELISAs for markers of gut permeability and endotoxemia</u>

Plasma concentrations of proteins of interest were in human and murine plasma using ELISA kits following the manufacturers' instructions. Details of ELISA kits utilized are available in ESM Table 4.

Western Blot

Antibodies used in western blot studies can be found in ESM Table 5.

Tissue lysates (cytoplasmic and nuclear fractions) were prepared from flash-frozen small intestine (jejunum and ileum) from all cohorts using the Cell Fractionation Kit purchased from Cell Signaling (9038) following the manufacturer's instructions. Protease inhibitors were used to prevent protein degradation and were supplied with the cell fraction kit. Protein content in the samples were measured using a colorimetric BCA protein assay purchased from Thermofisher Scientific (23225). For western blot analysis, the proteins (60–80µg) were resolved using 8–12% SDS-PAGE gels (Bio-Rad, Hercules, CA, USA) and transferred onto a nitrocellulose membrane (0.2µm). Nitrocellulose membranes were then incubated in the blocking buffer (5% BSA in TBST containing 0.05% Tween-20) to block the non-specific binding sites. Then, the membrane was incubated with the protein-specific primary antibody (1/500 dilution) overnight at 4°C. TBST was used to wash the membrane (5 min each, 3 times) to remove excess antibody. The membrane was then incubated with the appropriate peroxidase-conjugated secondary antibody (1/1000 dilution) at room temperature for 2h while rocking, followed by washing with TBST (5 min each, 3 times). Protein bands were visualized using chemiluminescence reagents. The membrane was stripped and re-probed with β-actin antibody to confirm equal protein loading. Other protein expressions were identified using protein ladders as molecular weight markers. The intensity value for each band was normalized to its β-actin loading control before being analyzed.

Murine neural retina flat-mount preparation, VE-cadherin staining, and acellular capillaries

Preparation: Eyes were enucleated, cleaned of fat and muscle, and the lens, vitreous, and cornea were carefully removed. Four shallow incisions were then made in the eyeball, allowing for separation of the retina from the RPE/choroid with minimal cross-contamination. The retina was then flattened on a glass slide with the RPE facing upward and gently covered with a glass coverslip after the staining.

Immunostaining of VE-Cadherin: Retina flat mounts were washed and then incubated with 0.1% Triton 100X, 15min. Then in blocking serum solution (5% NGS+1% BSA). For staining, anti-Mouse VE-cadherin Antibody (BV9), sc-52751, 1:200, was added and incubated overnight at 4°C followed by 1 hour 37°C. After washing the tissues, the following day, the secondary antibody, Alexa® Fluor-labeled (594 nm) goat anti-mouse IgG, 1:500 (Molecular Probes, Eugene, OR) was added and incubated at room temperature for 1 hour. Samples were washed, and anti-fade reagent (Molecular Probes) was added. Images were acquired within 24 hours using a Zeiss AxioImager.A2 upright fluorescence microscope.

Acellular capillary preparation: Eyes were fixed in 4% buffered formalin. Posterior eye cups were obtained by making a small incision through the limbus and dissecting out the cornea and lens. These posterior cups were floated in distilled water to separate the retina from the sclera. After careful removal of the sclera, retinas were left overnight in phosphate-buffered saline for unfixing. On the second day, retinas were digested in 3% Elastase (Difco, Oxford, UK) for 1.5–2 h, and, after careful removal of internal limiting membranes, retinas were allowed to stand in 0.5% Triton X−100 solution until the complete removal of the neural retina. Preparations were mounted on pre-sialinised slides and stained with light green (Fisher Scientific, Loughborough, Leics, UK) followed by counterstaining with haematoxylin (Sigma, Gillingham, Dorset, UK). Finally, slides were mounted in DPX mounting medium (Fisher Scientific), and acellular capillaries counted at 20× magnification in six random fields in the central retina in a masked fashion.

References:

[1] Bugger H, Boudina S, Hu XX, et al. (2008) Type 1 diabetic akita mouse hearts are insulin sensitive but manifest structurally abnormal mitochondria that remain coupled despite increased uncoupling protein 3. Diabetes 57(11): 2924-2932. 10.2337/db08-0079
[2] Duan Y, Prasad R, Feng D, et al. (2019) Bone Marrow-Derived Cells Restore Functional Integrity of the Gut Epithelial and Vascular Barriers in a Model of Diabetes and ACE2 Deficiency. Circ Res 125(11): 969-988. 10.1161/CIRCRESAHA.119.315743
[3] Prasad R, Floyd JL, Dupont M, et al. (2023) Maintenance of Enteral ACE2 Prevents Diabetic Retinopathy in Type 1 Diabetes. Circ Res 132(1): e1-e21. 10.1161/CIRCRESAHA.122.322003
[4] Leger H, Santana E, Beltran WA, Luca FC (2019) Preparation of Mouse Retinal Cryo-sections for Immunohistochemistry. J Vis Exp(149). 10.3791/59683
[5] Asare-Bediako B, Noothi SK, Li Calzi S, et al. (2020) Characterizing the Retinal Phenotype in the High-Fat Diet and Western Diet Mouse Models of Prediabetes. Cells 9(2). 10.3390/cells9020464

ESM Table 1: Antibodies utilized in flow cytometry studies.

Target	Clone	Source	Catalog #	Working dilution (μL)
Human				
CD45	HI30	BD Biosciences	566041	1:200
CD15	HI98	BD Biosciences	561584	1:200
Mouse				
CD45	30-F11	BD Biosciences USA	751170	1:200
c-Kit (CD117)	2B8	Invitrogen USA	48-1171-82	1:200
FcγRII/III (CD16/32)	93	Invitrogen USA	63-0161-82	1:200
Sca-1	D7	Invitrogen USA	64-5981-82	1:200
Hematopoietic Lineage (Lin) (CD3, CD45R, CD11b, TER-119, Gr-1)		Invitrogen USA	22-7770-72	1:200
Flt3	BV10A4H2	Invitrogen USA	17-1357-41	1:200
CD34	MEC14.7	Invitrogen USA	MA5-17831	1:200
Ly6G	1A8	BioLegend USA	127639	1:200
Ly6C	HK1.4	Invitrogen USA	47-5932-82	1:200
CD11b	M1/70	Invitrogen USA	78-0112-82	1:200
CCR2	SA203G11	BD Biosciences USA	150608	1:200
Flk-1	Avas12a1	BD Biosciences USA	560681	1:200
CD31	390	Invitrogen USA	12-0311-82	1:200
CD206	MR6F3	Invitrogen USA	17-2061-82	1:200
F4/80	BM8	Invitrogen USA	48-4801-82	1:200
CD107a	1D4B	BD Biosciences USA	565533	1:200
CD62L	MEL14	BD Biosciences USA	612833	1:200
Gr-1	RB6-8C5	BD Biosciences USA	552093	1:200
CXCR2	SA044G4	BD Biosciences USA	149312	1:200
CXCR4	2B11	Invitrogen USA	12-9991-82	1:200
CD63	NVG-2	BD Biosciences USA	143908	1:200

ESM Table 2: Primers used in mouse genotyping.

Gene	Primer sequences		Product size
Akita	Forward (5'-3')	TGC TGA TGC CCT GGC CTG CT	Mutant: 280bp WT: 140bp
	Reverse (5'-3')	TGG TCC CAC ATA TGC ACA TG	
db/db	Forward (5'-3')	AGA ACG GAC ACT CTT TGA AGT CTC	Mutant: 108 WT: 135bp
	Common (5'-3')	CAT TCA AAC CAT AGT TTA GGT TTG TGT	

ESM Table 3. Antibodies used in immunofluorescence studies.

Target	Clone	Source	Catalog #	Working dilution (μL)
Primary				
Ly6G-CF405M	RB6-8C5	Abcam USA	ab236571	1:200
NIMP-R14		Santa Cruz USA	sc59338	1:200
Cit-H3	polyclonal	Abcam USA	ab5103	1:200
Collagen IV	polyclonal	Abcam USA	ab19808	1:200
ZO-1	R40.76	Santa Cruz USA	sc33725	1:200
p120-Catenin	D7S2M	Cell Signaling USA	59854S	1:200
E-Cadherin	24E10	Cell Signaling USA	3195S	1:200
VE-Cadherin (intestinal)	polyclonal	Abcam USA	ab33168	1:200
PV-1	MECA-32	Santa Cruz USA	sc-19603	1:200
IDO1	polyclonal	LS Bio USA	LS-B1746-50	1:200
VE-Cadherin (retinal)	BV9	Santa Cruz USA	sc-52751	1:200
Secondary				
Donkey anti-Rabbit IgG (H+L) – Alexa Fluor 405		Invitrogen USA	A48258	1:500
Goat anti-Rabbit IgG (H+L) – Alexa Fluor 488		Invitrogen USA	A11034	1:500
Goat anti-Rabbit IgG (H+L) – Alexa Fluor 594		Invitrogen USA	A11037	1:500
Goat anti-Rat IgG (H+L) – Alexa Fluor 488		Invitrogen USA	A11006	1:500
Goat anti-Rat IgG (H+L) – Alexa Fluor 594		Invitrogen USA	A11007	1:500
Donkey anti-Goat IgG (H+L) - Alexa Fluor 594		Invitrogen USA	A11058	1:500
Goat anti-Mouse IgG (H+L) Alexa Fluor 594		Invitrogen USA	A11005	1:500

ESM Table 4. Enzyme-Linked Immunosorbent Assay (ELISA) Kits.

Target	Source	Catalog #
Human		
FABP2	Abcam USA	ab234566
PGN	MyBioSource USA	MBS285961
LBP	Abcam USA	ab279407
Mouse		

FABP2	MyBioSource USA	MBS161452
PGN	MyBioSource USA	MBS263268
NE	Abcam USA	ab252356

ESM Table 5. Antibodies used in western blot studies.

Target	Clone	Source	Catalog #	Working dilution (µL)
Primary				
E-Cadherin	24E10	Cell Signaling USA	3195S	1:500
PV-1	MECA-32	Novus Biologicals USA	nb77668	1:500
β-Actin-HRP	AC-15	Sigma USA	A3854	1:10000
Secondary				
Mouse anti-rabbit IgG-HRP		Santa Cruz USA	sc-2357	1:1000
Goat anti-rat IgG-HRP		Cell Signaling USA	7077S	1:1000

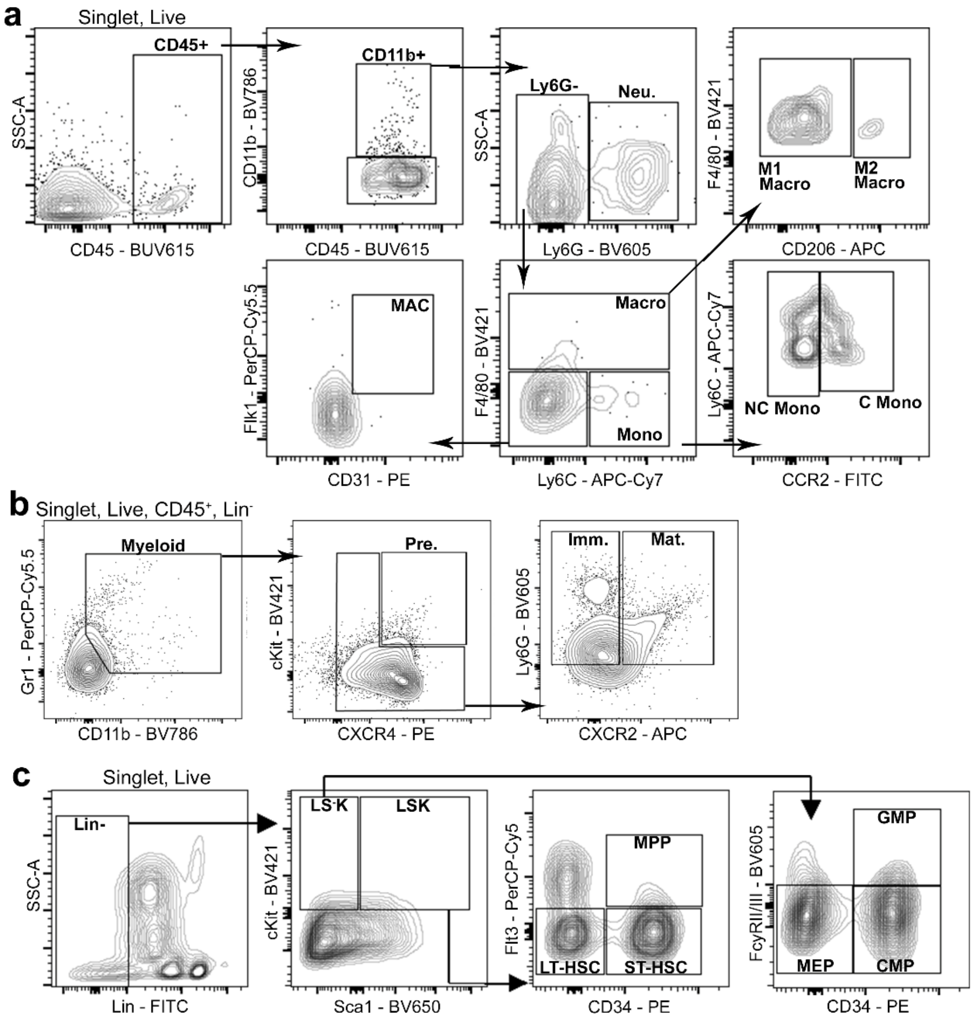

ESM Figure 1. Flow cytometry gating strategies. (a) Gating strategy of myeloid cell population. (b) Gating strategy of neutrophil subpopulations. A lineage cocktail of FITC-conjugated antibodies was included with neutrophil-relevant antibodies to perform negative selection of neutrophils by exclusion of T-cells, B-cells, NK cells, monocytes, macrophages, megakaryocytes, platelets, and erythrocytes (CD3ε, CD161, CD90.2, B220, Siglec-F, CD115, and F4/80). (c) Gating strategy of hematopoietic stem and progenitor cells. Lineage cocktail included markers for lineage-committed cells of the myeloid (CD11b, Gr-1), erythroid (TER-119), and lymphoid (CD3, CD45R) lineages.

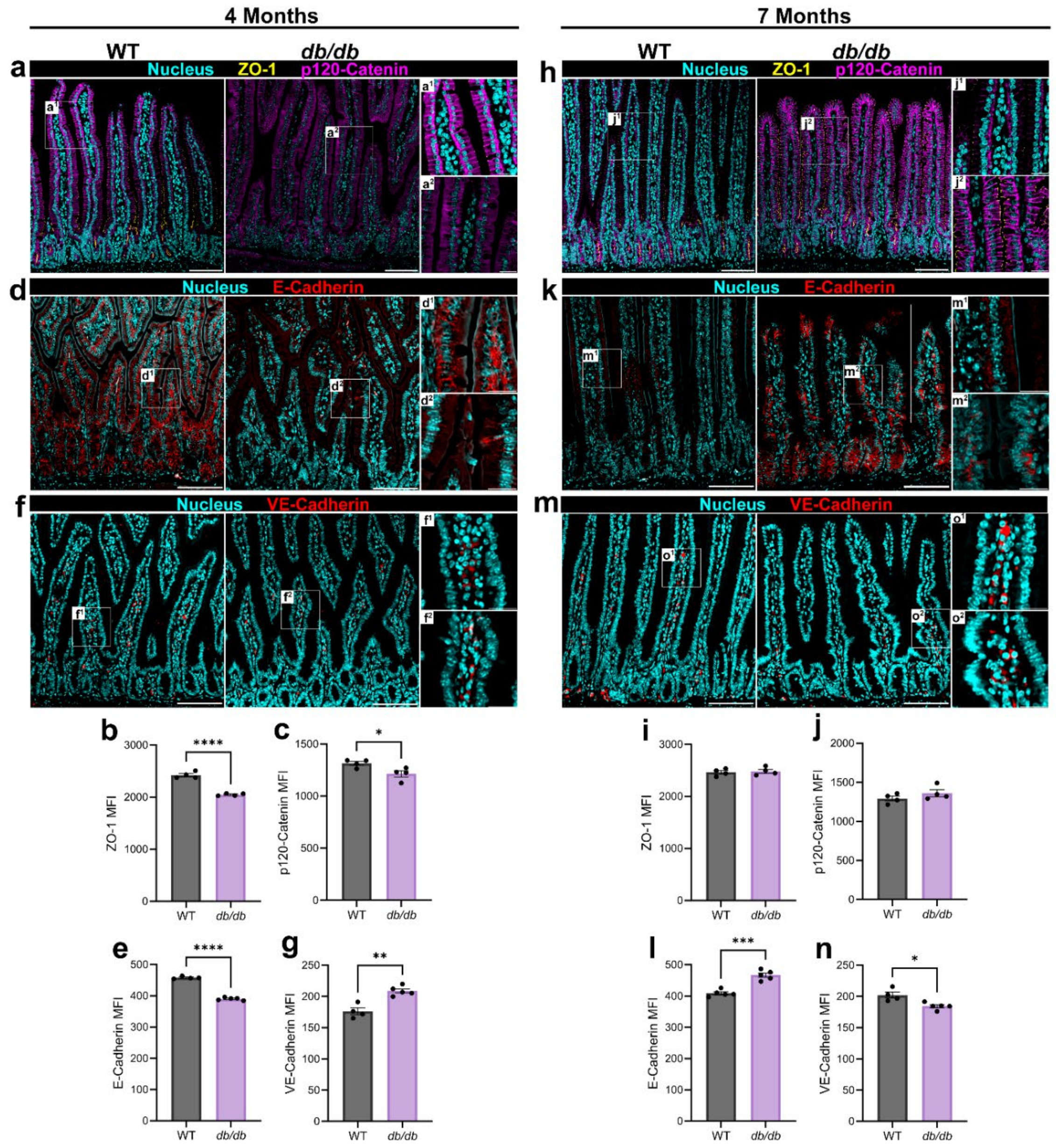

ESM Figure 2. NETosis is associated with compensatory dysfunction of the gut epithelial and endothelial barriers. To assess whether a hyperpermeable gut barrier is associated with elevated NETosis, we performed confocal IF microscopy of FFPE small intestine tissue sections and plasma ELISAs of permeability markers in *db/db* mice with (a-g) 4 and (h-n) 7 months of T2D duration and age-matched WT mice. Representative images (A) ZO-1 and p120-Catenin, (d) E-cadherin, and (f) VE-cadherin expression at 4 months. Quantifications of (b) ZO-1, (c) p120-Catenin, (e) E-Cadherin, and (g) VE-Cadherin MFI at 4 months. Representative images (h) ZO-1 and p120-Catenin, (k) E-cadherin, and (m) VE-cadherin expression at 7 months. Quantifications of (i) ZO-1, (j) p120-Catenin, (l) E-Cadherin, and (n) VE-Cadherin MFI at 7 months. Each black dot represents the average of 3-4 randomly dispersed images taken from the tissue of one experimental animal (n=3-5 animals per group). Data which does not include an indication of statistical significance was found to be non-significantly altered. Scale bars: low power = 100μm, high power = 25μm

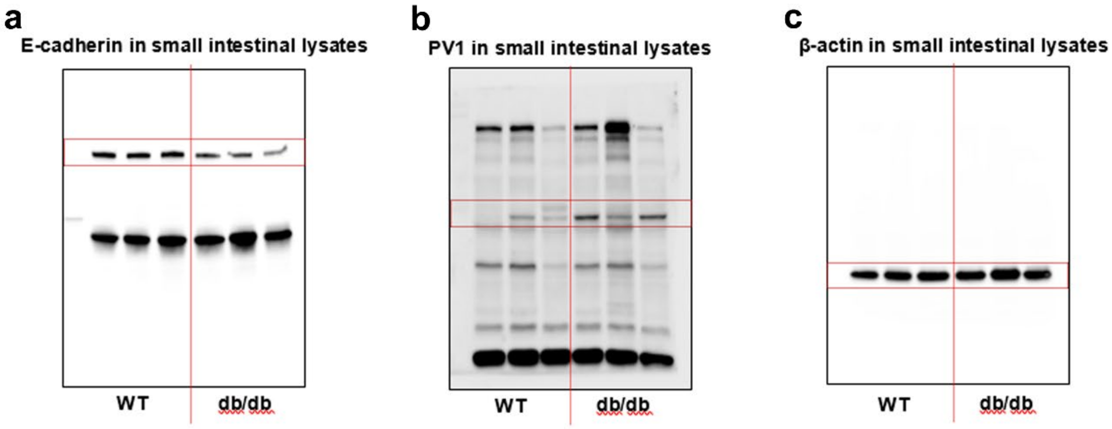

ESM Figure 3. Full uncropped immunoblots of E-cadherin and PV1 and their loading control (β-Actin) used in Figure 4. The red-dotted rectangle indicates the portion of the blot displayed in the manuscript.

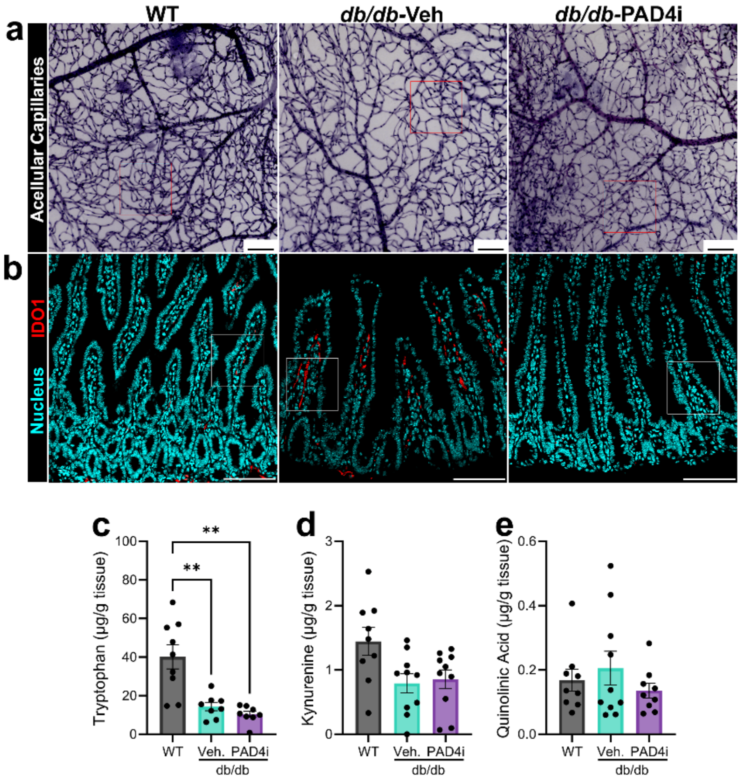

ESM Figure 4. PAD4i-induced alterations to intestinal tryptophan metabolism. Representative low magnification images of (a) retinal acellular capillaries and (b) intestinal IDO. Concentrations of (c) Tryptophan, (d) Kynurenine, and (e) Quinolinic Acid within small intestine tissue as determined by mass spectrometry. Data which does not include an indication of statistical significance was found to be non-significantly altered. Scale bars = 100µm.

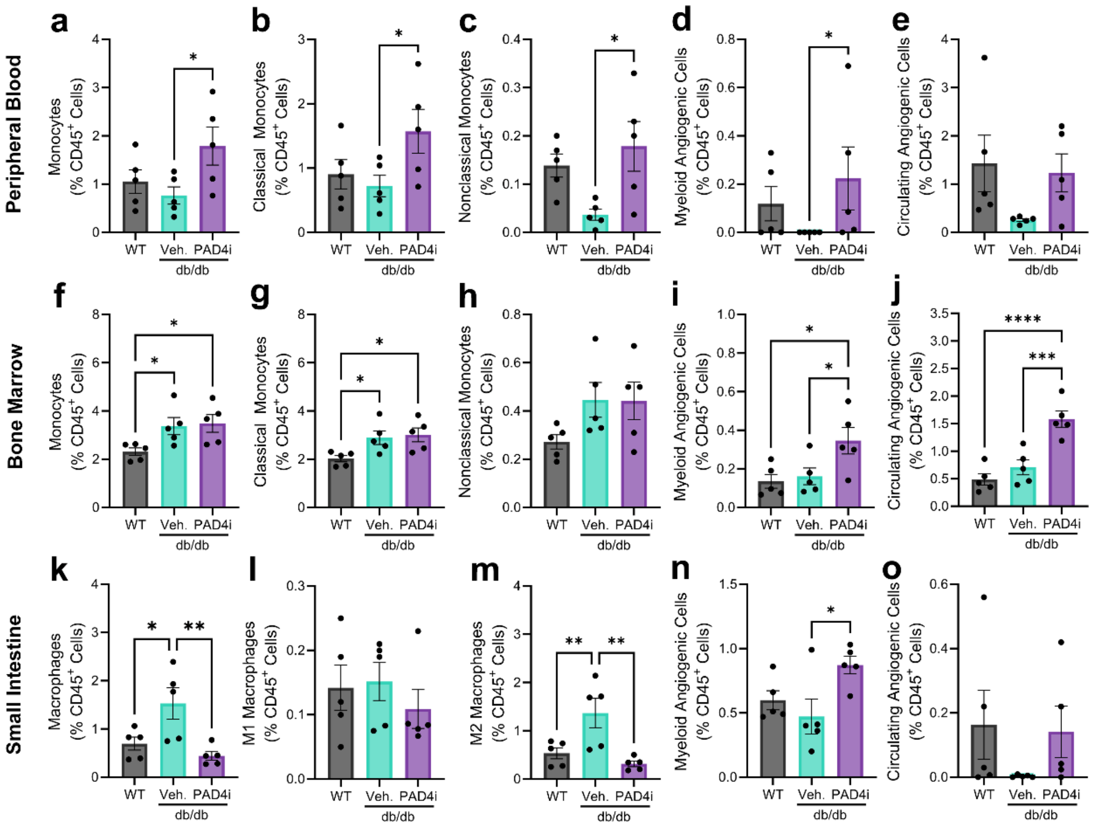

ESM Figure 5. PAD4i-induced alterations of the myeloid and angiogenic immune compartments. The proportions of relevant immune cell populations were assessed by flow cytometry within the peripheral blood, bone marrow, and small intestine. Quantification of peripheral blood (a) total monocytes, (b) classical monocytes, (c) nonclassical monocytes, (d) myeloid angiogenic cells (MACs), and (e) non-myeloid circulating angiogenic cells (CACs). This pattern was replicated for the (f-j) bone marrow and (k-o) small intestine. Each black dot represents one independent observation from one experimental animal (n=5 per group). Data which does not include an indication of statistical significance was found to be non-significantly altered.

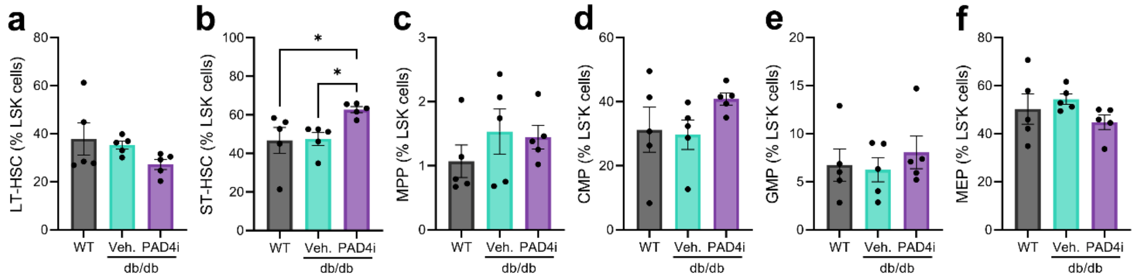

ESM Figure 6. PAD4i induces negligible effects on hematopoietic stem and progenitor cells. The proportions of bone marrow HSPCs were assessed by flow cytometry. Quantifications of (a) long-term repopulating hematopoietic stem cells (LT-HSC), (b) short-term repopulating hematopoietic stem cells (ST-HSC), (c) multipotent progenitor cells (MPP), (d) common myeloid progenitors (CMP), (e) granulocyte-monocyte progenitors (GMP), and (f) megakaryocyte-erythroid progenitors (MEP). Each black dot represents one independent observation from one experimental animal (n=5 per group). Data which does not include an indication of statistical significance was found to be non-significantly altered.
